# Association between exposure to greenness and atopic march in children and adults—A systematic review and meta-analysis

**DOI:** 10.3389/fpubh.2022.1097486

**Published:** 2023-01-09

**Authors:** Xue Wang, Nan Zhou, Yuxiang Zhi

**Affiliations:** Department of Allergy and Clinical Immunology, Peking Union Medical College Hospital, Peking Union Medical College and Chinese Academy of Medical Sciences, National Clinical Research Center for Immunologic Diseases, Beijing, China

**Keywords:** allergic disease, atopic march, greenness exposure, NDVI, asthma

## Abstract

**Introduction:**

Allergic diseases are a global public health problem. Food allergy, atopic dermatitis (AD), allergic rhinoconjunctivitis, allergic rhinitis (AR) and asthma represent the natural course of allergic diseases, also known as the “atopic march”. In recent years, a large number of studies have been published on the association between greenness exposure and allergic diseases. However, systematic reviews on the association between greenness exposure and multiple allergic diseases or atopic march are lacking.

**Methods:**

In this study, PubMed, EMBASE, ISI Web of Science, and Scopus were systematically searched. Meta-analyses were performed if at least three studies reported risk estimates for the same outcome and exposure measures.

**Results:**

Of 2355 records, 48 studies were included for qualitative review. Five birth cohort studies, five cross-sectional studies, and one case-control study were included for asthma meta-analysis, respectively. Four birth cohort studies were included for AR meta-analysis. Our results support that exposure to a greener environment at birth reduces the risk of asthma and AR in childhood. In addition, higher greenness exposure was associated with decreased odds of current asthma in children.

**Discussion:**

There was a large heterogeneity among the included studies and most of them did not specify the vegetation type and causative allergens. Therefore the study results need to be further validated. In addition, a small number of studies evaluated the association between greenness and food allergy, AD and allergic rhinoconjunctivitis. More research is needed to strengthen our understanding of the association between greenness and allergic diseases.

## Introduction

Allergic diseases have become a global public health problem (1). Asthma, allergic rhinitis (AR), atopic dermatitis (AD), and food allergy have been reported to affect 30% of the population and nearly 80% of households (1). Allergic diseases have very complex pathophysiological mechanisms and a strong genetic component (2), but genetic changes alone cannot explain the rapid increase in prevalence over the last 50 years (3, 4). A large number of epidemiological studies have demonstrated complex gene-environment interactions, highlighting environmental exposure as an important factor influencing the phenotype of allergic diseases (3, 5). Increased exposure to greenness is one such factor.

Greenness is a combination of plant species and the ground cover they provide. It can be either scheduled (e.g., gardens and parks) or unscheduled (e.g., existing wooded areas and prairies) (6). In recent years, greenness has been shown to improve physical activity (7), mental health (8), birth outcomes (9), cardiovascular disease (10), obesity (11), diabetes (12), and cancer (13). Although not all studies show consistent findings, overall, the available studies support the benefits of greenness for human health. It is generally accepted that greenness may affect health by providing a greater number and diversity of beneficial microorganisms (14), which contribute to relieving mental and physiologic stress, promoting exercise and socialization, as well as reducing exposures to heat, noise, and air pollution (15).

In recent years, a number of studies on the effects of greenness on several allergic diseases have been published, indicating a growing interest in greenness and allergies. However, research findings are highly inconsistent, even within the same study. Fuertes et al. reported that greenness within the 500 m buffer around the home address (accessible within a short walking time) was a risk factor for AR at age 6–8 years in Swedish (BAMSE) and German (GINI/LISA South), but was a protective factor in German (GINI/ LISA North) and Dutch (PIAMA) (16). Rufo et al. (17) reported that living closer to a green environment at birth had a protective effect on allergic diseases and asthma at age 7 years, while this effect was not significant at age 4 years. Sbihi et al. (18) reported that perinatal exposure to greenness within a 100 m buffer around the home address (the most accessible greenness) was associated with a reduced incidence of asthma. However, Andrusaityte et al. (19) reported that increased greenness within a 100 m buffer around the home address significantly increased the risk of asthma.

A meta-analysis conducted by Lambert et al. (20) reviewed the association between greenness and allergic respiratory diseases in children and adolescents. They reported no significant overall association between residential greenness and asthma or AR. Since this review, a large number of relevant studies have been published, which have enriched the understanding of the relationship between greenness, asthma, and AR. In addition, recent studies have begun to focus on the effects of greenness exposure on AD, food allergy, and allergic rhinoconjunctivitis. These allergic diseases may occur in a chronological order: from AD and food allergy in infancy to asthma and AR in childhood. This phenomenon is known as “atopic march.” To our knowledge, there is no meta-analysis of the most recent evidence on the association between greenness, various allergic outcomes, and atopic march. Therefore, the aim of this study was to provide a systematic review of the available evidence to elucidate the association between greenness exposure and allergic diseases based on the Preferred Reporting Items for Systematic Reviews and Meta Analyses (PRISMA) statement (Supplementary Table S1). This may help urban planners and policy makers to better plan urban layouts and thus reduce the risk of allergic diseases.

## Methods

### Literature search strategy

The PubMed (https://pubmed.ncbi.nlm.nih.gov/), ISI Web of Science (http://www.webofknowledge.com), EMBASE (https://www.embase.com), and Scopus (https://www.scopus.com/) databases were systematically searched for English language, peer-reviewed studies published up to 30 November 2022. Unpublished and ongoing studies were not considered in this review. We used a combination of terms concerning greenness exposure (“greenspace,” “green space,” “greenness,” “greenery,” “grassland,” “grass land,” “green land,” “grass cover,” “natural area,” and “vegetation”) and allergic outcomes (“allergy,” “allergic disease,” “asthma,” “hay fever,” “allergic rhinitis,” “allergic respiratory diseases,” “eczema,” “atopic dermatitis,” “food allergy,” and “atopic march”) for the search (Supplementary Table S2). No filters were applied to the study designs. The reference lists of the included studies were manually searched to identify any remaining studies.

### Inclusion and exclusion criteria

Original studies assessing the relationship between greenness and allergic disease were included. For eligible studies, the definition of allergic outcomes should be clearly stated (medical records, structured interviews, or self-reported physician-diagnosed allergic outcomes). The assessment of greenness exposure should be objective. Assessment methods of greenness include, but are not limited to, the normalized difference vegetation index (NDVI), light detection and ranging (LiDAR) imagery, and distance to the nearest greenness. Eligible articles are also required to report the association of greenness with allergic disease. Odds ratios (OR), relative risks (RR), or hazard ratios (HR), and their 95% confidence intervals (CI) should be provided. Studies with only allergic symptoms (e.g., wheezing, dry cough, and symptom scores), lung function, and sensitization (e.g., immunoglobulin E antibody levels and skin prick tests) as outcomes were not considered in this review.

### Study selection

All potentially relevant articles were exported to EndNote 20.2 (Clarivate Analytics, Philadelphia, PA, USA). Duplicate articles were removed. Titles and abstracts were screened to eliminate articles that were obviously irrelevant. The full text of relevant articles was downloaded and read in depth. All processes were performed independently by two authors (XW and NZ). Disagreements were resolved through discussion with the third author (YZ).

### Data extraction

For each eligible study, the required information was extracted independently by two authors (XW and NZ). Discrepancies were resolved by discussion with the third author (YZ). The following information was extracted: authors, publication year, study period, study location, study design, participant demographics, sample size, assessment of greenness exposure, definition of allergic outcomes, covariates adjusted for in the statistical model, and effect estimates (OR, RR, or HR, and their 95% CI). Effect estimates were extracted in the “main model” or “fully variable adjusted model” (21). If the results in the original study were presented as figures, Engauge Digitizer 12.1 was used to extract the data.

### Quality assessment of included studies

The quality of each eligible study was assessed independently by two authors (XW and NZ). Discrepancies were resolved by discussion with the third author (YZ). The Newcastle-Ottawa scale (NOS) was employed to assess the quality of cohort and case-control studies (22). The NOS contains eight items grouped into three dimensions. Each item is scored with 0 or 1 star, except for one item (comparability of cohorts on the basis of the design or analysis) that is scored with 0 to 2 stars, resulting in a maximum score of 9 stars. We classified studies with scores >7 stars as “high quality”; otherwise, the study was classified as “low quality.” The Quality Assessment Tool for Observational Cohort and Cross-Sectional Studies was used to evaluate the quality of cross-sectional studies (23). This tool consists of 14 questions, but only seven questions applicable to cross-sectional studies were used (24). Studies were rated as poor, fair, or good; if the study did not adjust for any variables, it was rated as poor.

### Meta-analysis methods

Given that multiple allergic diseases were included as outcomes in this review, three studies measuring the same outcome with the same exposure was selected as thresholds in order to decide whether to perform a meta-analysis on a particular outcome. That is, meta-analysis was performed when the number of studies meeting the following conditions was greater than or equal to three: (1) same assessment of greenness exposure, including measurement methods, measurement locations (residential, school), and time windows (for birth cohort: at birth/perinatal or lifetime); (2) same outcome definitions, including ever asthma, current asthma, AR, allergic rhinoconjunctivitis, AD, and food allergy; and (3) similar age of participants, including minors [preschoolers (<5 years old) and post-schoolers (5–18 years old) if a sufficient number of studies were available], and adults (>18 years old). To ensure consistency in the interpretation of the original study results, only studies that used NDVI as a measure of greenness were included in the meta-analysis, as NDVI was used in the majority of the included studies.

Based on the results of the included studies, two independent types of meta-analyses were carried out. In the dose-response meta-analysis, we used adjusted OR (95% CI) per 0.1 unit increase in greenness exposure measured by NDVI. In the categorical meta-analysis, we compared the highest tertile or quartile (Q3 or Q4) with the lowest (Q1). All studies included in the meta-analysis reported OR. Therefore, OR was used as the measure of association across all studies.

Fixed-effect and random-effect models were used to synthesize the associations between greenness exposures and allergic diseases. The between-study heterogeneity and between-study variance were assessed using *I*^2^ and τ^2^, respectively. Egger's test and Begg's test were used to assess the publication bias. In addition, since different buffers represent different accessibility to greenness, a 100 m buffer represents the most accessible range of greenness, while a 500 m buffer represents the availability of greenness within a relatively short walking time (i.e., <15 mins). Therefore, subgroup analyses were performed based on the buffer zones. The results of 100 m, more than 100 m and <500 m, 500 m, and more than 500 m buffers were combined, respectively. To test the robustness of the result, sensitivity analyses were performed by excluding studies one by one. All analyses were performed with R software (R Foundation for Statistical Computing), version 3.5.0. A two-sided test was used, and *p* < 0.05 was considered as statistically significant.

## Results

**Figure 1:**
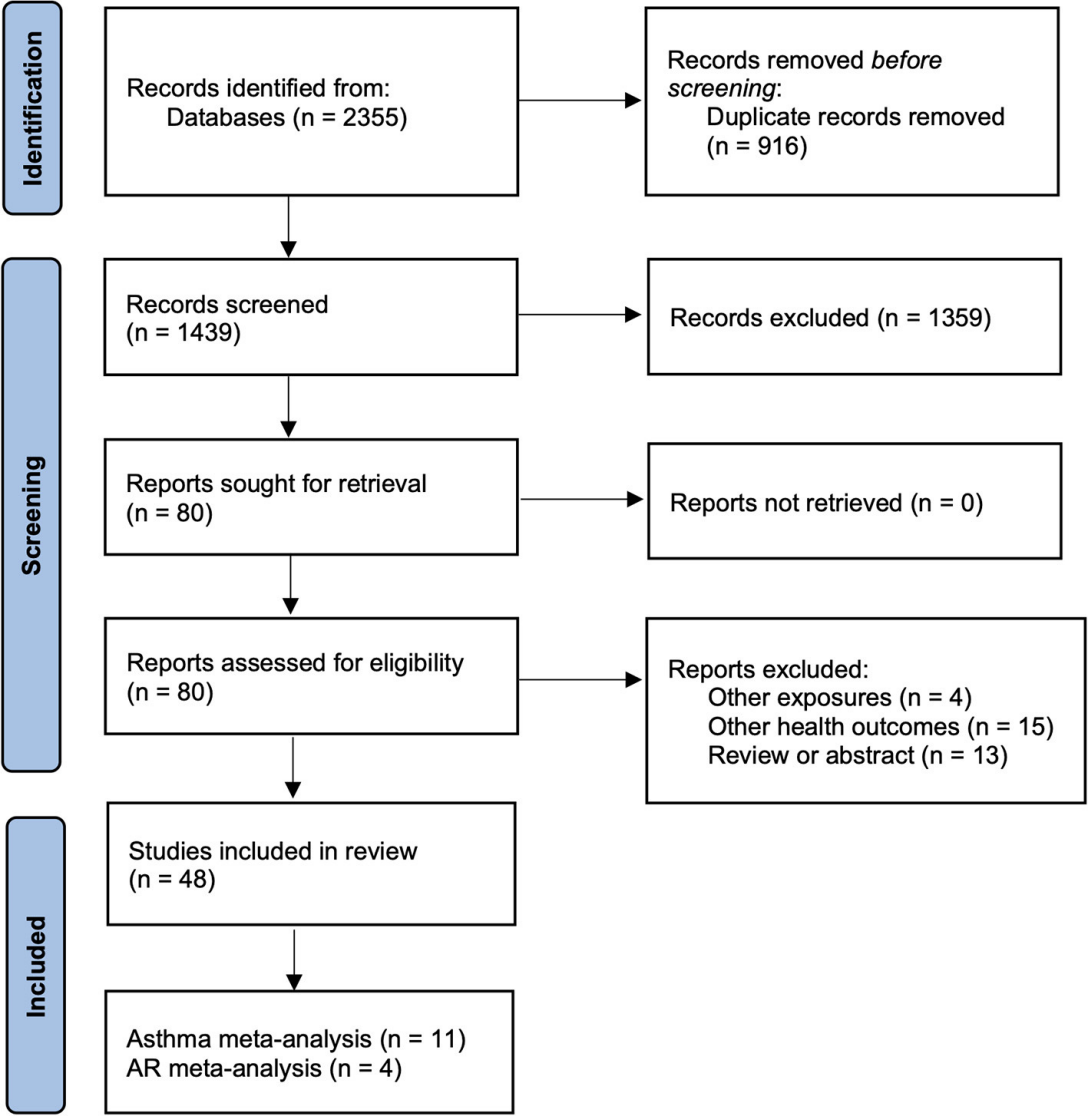
PRISMA flowchart for search strategy.

The electronic search strategy generated 2,355 records, which were screened for eligibility based on title/abstract after de-duplication. 80 potentially eligible articles were selected for full-text review. Of these, 32 articles were excluded because they did not assess the relationship between greenness exposure and allergic disease. Finally, 48 articles fulfilled the inclusion criteria and were included in the qualitative synthesis (Figure 1).

### Study characteristics

Table 1 summarizes the characteristics of the 48 studies included in the review. More than half of the studies (*n* = 25) were published within the past 3 years and almost all (*n* = 47) were published within the past 10 years, highlighting the growing interest in the association between greenness and allergic diseases. Of the 48 studies, 47 were conducted in more than 20 countries and one study was conducted globally, reflecting the association of greenness with allergic disease in a global context. There were 19 cohort or longitudinal studies, 18 cross-sectional studies, 7 ecological studies, and 4 case-control studies. Supplementary Table S3 shows more detailed information for each study, including exposure definitions, outcome definitions, statistical models, estimates, and adjusted confounders.

**Table 1:** Characteristics of the included studies.

**References**	**Years of study**	**Location**	**Study design**	**Age of participants**	**Sample size (n)**	**Exposure measure**	**Allergic diseases assessed**				
							**Asthma**	**AR**	**Allergic rhino conjunctivitis**	**AD**	**Food allergy**
Alcock et al. (25)	1997–2012	England	Cross-sectional	No age limit	26,455	Generalized land use database; ArcGIS	✓				
Andrusaityte et al. (19)	2012–2013	Lithuania	Nested case-control	4–6	1,489	NDVI and distance to the nearest city park	✓				
Brokamp et al. (26)	Ongoing	The United States	Birth cohort	Children born in 2001–2003	762	NDVI	✓				
Cilluffo et al. (27)	2015–2018	Italy	Longitudinal study	5–16	179	NDVI	✓				
Dadvand et al. (28)	2006	Spain	Cross-sectional	9–12	3,178	NDVI and residential proximity to green spaces	✓		✓		
De Roos et al. (29)	2011–2016	The United States	Case-control	< 18	23,382	NDVI, LiDAR	✓				
DePriest et al. (30)	–	The United States	Cross-sectional	< 18	196	NDVI	✓				
Dong et al. (31)	–	Canada	Cross-sectional	All ages		LiDAR	✓				
Donovan et al. (32)	1998–2016	New Zealand	Birth cohort	Children born in 1998 and followed up until 2016	49,956	NDVI; the total number of natural land-cover types	✓				
Donovan et al. (33)	–	The United States	Ecological	>18	498 cities, 26,367 tracts	NDVI; plant diversity	✓				
Douglas et al. (34)	–	The United States	Ecological	No age limit	2,347 census tracts	Public parks and open space (PPOS)	✓				
Dzhambov et al. (35)	2004–2005	Austria and Italy	Cross-sectional	8–12	1,251	NDVI, tree canopy cover, and agricultural cover	✓	✓		✓	
Eldeirawi et al. (36)	2004–2005	The United States	Cross-sectional	8–18	1,915	NDVI	✓				
Fuertes et al. (37)	GINIplus: 1995; LISAplus: 1997	Germany	Birth cohort	–	5,803	NDVI		✓			
Fuertes et al. (38)	2000–2003	Global	Global ecologic analysis	6–7; 13–14	1,002,617	NDVI		✓			
Fuertes et al. (16)	Recruited in 1990–1997	Sweden, Australia, Netherlands, Canada, Germany	Birth cohort	6–8; 10–12	13,016	NDVI		✓			
Gernes et al. (39)	2001–2003	The United States	Birth cohort	–	478	NDVI and LiDAR		✓			
Hartley et al. (40)	2001–2003	The United States	Birth cohort	–	617	NDVI	✓				
Hsieh et al. (41)	2001–2013	Taiwan, China	Case-control	Younger than 18 years	7,040	NDVI	✓				
Hu et al. (42)	2019	China	Cross-sectional	3–12	16,605	NDVI and EVI	✓				
Idani et al. (43)	2017–2018	Iran	Cross-sectional	20–65	5,672	Questionnaire	✓				
Ihlebæk et al. (44)	2000–2001	Norway	Cross-sectional	29, 30, 39 40, 44, 45, 58, 59 60	8,638	Vegetation cover greenness (VCG) and land use greenness (LUG)	✓				
Kim et al. (45)	2009	Korea	Cross-sectional	49.37 ± 16.24	219,298	Green areas		✓		✓	
Kim and Ahn (46)	2011–2013	The United States	Ecological			LiDAR	✓				
Kuiper et al. (47)	2013–2015	Northern Europe, Spain, Australia	Generation study	Parents: mean age 35; offspring: mean age 6	1,106 parents with 1,949 offspring	NDVI	✓	✓			
Kuiper et al. (48)	2013–2015	Norway, Sweden	Case-control	Mean age 28	3,428	NDVI	✓	✓			
Kwon et al. (49)	2014	Korea	Cross-sectional	>20	10.5 million	NDVI		✓			
Lee et al. (50)	2006–2010	Korea	Birth cohort	Outcomes were evaluated at 6 months	659	Land cover classification maps				✓	
Lee et al. (51)	2003–2011	Taiwan, China	Cohort study	Children diagnosed with AR at age 4 were followed up to age 12	11,281	NDVI		✓			
Lee et al. (52)	2010–2014	The United States	Ecological	Aged 19 years old or below	763 census tracts	Land cover database	✓				
Li et al. (53)	2014–2015	China	Cross-sectional	Mostly between the ages of 12 and 15	5,643	NDVI and distance to the nearest park	✓	✓		✓	
Lin et al. (54)	Baseline (2017–2018)	China	Birth cohort	2	522 mother–child pairs	NDVI, enhanced vegetation index (EVI)		✓		✓	✓
Lovasi et al. (55)	–	The United States	Cross-sectional	< 15	Unknown	Street tree density	✓				
Lovasi et al. (56)	–	The United States	Birth cohort	Children born in 1998–2006	549	LiDAR	✓	✓			
Markevych et al. (57)	Neonates were recruited between 1997 and 1999	Germany	Birth cohort	2–15	631	NDVI	✓	✓			✓
Parmes et al. (58)	1991–2010	Italy, France, Slovenia and Poland	Ecological	3–14	8,063	Land cover classification maps	✓	✓		✓	
Peters et al. (59)	2007–2011	Australia	Wave 1 of the population-based HealthNuts study	12-month-old	5,276	NDVI					✓
Pilat et al. (60)	2005–2006	The United States	Ecological	< 17	–	NDVI	✓				
Putra et al. (61)	Recruited in 2004 and then followed-up biennially	Australia	Longitudinal study	2–15	9,589	Perceptions of green space quality	✓				
Cavaleiro Rufo et al. (17)	2005–2012	Portugal	Birth cohort	7	1,050	NDVI and SRI	✓	✓		✓	
Sbihi et al. (18)	1999–2009	Canada	Birth cohort	0–5; 6–10	51,857	NDVI	✓				
Sbihi et al. (62)	1999–2009	Canada	Birth cohort	0–10	65,254	NDVI	✓				
Squillacioti et al. (63)	2002–2010	Italy	Cross-sectional	10–13	126	NDVI	✓				
Tischer et al. (64)	Ongoing	Spain	Birth cohort	4	2,472	NDVI	✓	✓			
Winnicki et al. (65)	–	Denmark	Cohort study	Individuals born 1995–2015	40,249	Percentage greenspace	✓				
Wu et al. (66)	2006–2010	UK	Cross-sectional	37–73	351,717	NDVI	✓				
Yu et al. (67)	2009–2013	China	Cross-sectional	2–17	59,754	Green view index (GVI) and NDVI	✓				
Zeng et al. (68)	2012–2013	China	Cross-sectional	10.3 ± 3.6	59,754	NDVI, SAVI	✓				

### Study quality

In general, most cohort and cross-sectional studies were “high quality” or “good” (Supplementary Tables S4, S5). However, of the case-control studies, only one was rated as “high quality.” The other three case-control studies were rated 5 or 6 due to self-reported case definitions, hospital controls, and lack of comparability (Supplementary Table S6). A birth cohort study in Chinese toddlers identified allergic diseases based on medical records at age 2 years (54). However, AR usually requires several years of allergen exposure to develop. Accordingly, it is uncommon in children under 2 years of age. We therefore consider that the follow-up period of this study was not long enough for outcomes to occur (Supplementary Table S4).

### Bias due to outcome assessment

The definition of outcomes was based primarily on self/parent-reported questionnaires, hospital records, and medication prescriptions. Asthma was the most frequently measured clinical outcomes and were reported by 38 studies. Four studies simultaneously assessed the association between greenness, ever asthma, and current asthma (35, 48, 58, 67). One study used eosinophilic asthma as the outcome, defined as participants reporting asthma-related medication use and elevated eosinophil counts to at least 150 cells per μl (66). Five studies reported the association between greenness and asthma emergency department visits or hospitalization rates (25, 34, 46, 52, 55). AR was reported by 17 studies. One study classified AR as intermittent and persistent rhinitis (38). Two studies used the number of clinic visits for AR as the outcome (49, 51). AD, allergic rhinoconjunctivitis, and food allergy were reported in 7, 1 and 2 studies, respectively.

### Bias due to exposure assessment

The reviewed studies all used different methods to assess participants' exposure to greenness, including normalized difference vegetation index (NDVI), soil-adjusted vegetation index (SAVI) (68), green view index (GVI) (67), light detection and ranging (LiDAR) imagery (29, 31, 39, 46, 56), land cover classification maps (50, 58), and questionnaire (43), with varying buffer levels. The most common method is NDVI, which was used in 33 studies. NDVI is a satellite image-based vegetation index provided by the National Aeronautics and Space Administration (NASA) to measure greenness at the regional level (51), and has been validated for use in epidemiological studies (69). NDVI is calculated from the visible (red) and near-infrared light reflected by vegetation. NDVI values range between −1 and 1, with higher values corresponding to a higher density of healthy vegetation and lower values corresponding to barren rocky, sandy or snowy areas. Most studies used NDVI to assess greenness exposures at the individual-level, including residential greenness (16–19, 26–28, 35–37, 39–42, 47, 48, 51, 53, 54, 57, 59, 62–64, 66) and school greenness exposure (35, 67, 68). Studies have also evaluated NDVI values at the district-level and country-level (38, 49, 60). For birth cohorts, different exposure time windows (e.g., at birth, a certain age range, and lifetime) also contributed to the heterogeneity of studies. In addition, seasonal differences may have a significant influence on NDVI (20). Most studies used images taken during the most vegetated season of the year (spring/summer) to calculate NDVI, which was able to maximize the spatial contrast of greenness (16, 17, 19, 26, 28, 35–37, 39, 40, 47, 48, 57, 63, 67, 68). Two studies used images from spring, summer, fall and winter to calculate NDVI (51, 53). Some studies also used annual or seasonal median NDVI values to represent greenness (29, 42, 59). Overall, the diversity of exposure measurement makes it difficult to compare the effect estimates reported in these studies.

### Association between greenness exposure and asthma

**Figure 2:**
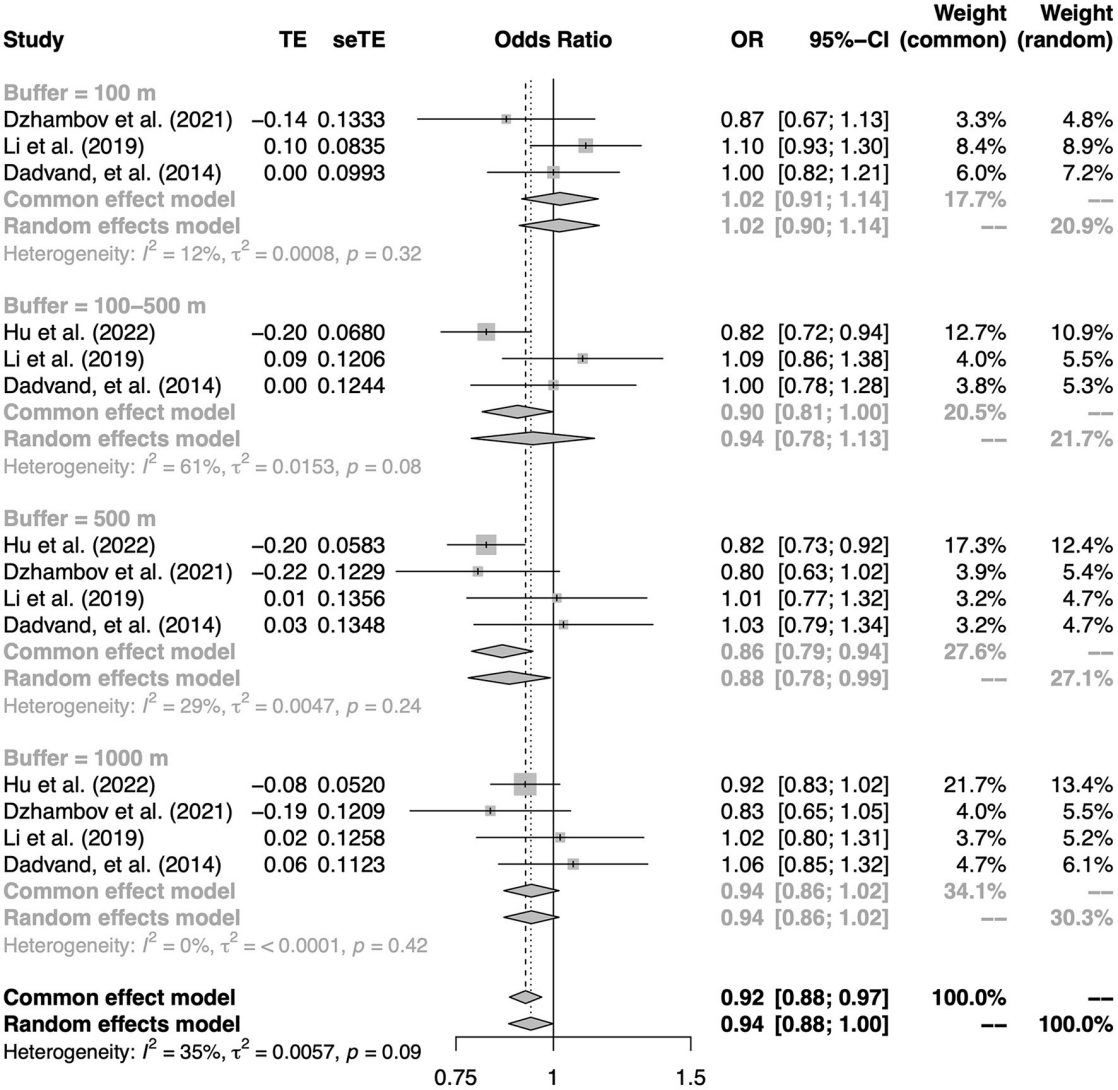
The association between residential greenness and current asthma. Subgroups were defined according to the buffers of the exposure measure.

Results of five cross-sectional studies and one case-control study reporting on the association between per IQR increase in NDVI and asthma were considered for quantitative synthesis. Two studies included both ever asthma and current asthma as outcomes (35, 53), two studies used ever asthma as an outcome (19, 36), and two study used current asthma as an outcome (28, 42). Quantitative analysis was performed separately for both outcomes. Figure 2 shows that an IQR increase in NDVI was associated with decreased odds of current asthma, with a pooled OR of 0.94 (95% CI: 0.88–1.00). However, no significant association was found between residential greenness exposure and ever asthma, regardless of buffer distance. The pooled OR of ever asthma per IQR increase in NDVI were 1.00 (95% CI: 0.93–1.08, Supplementary Figure S1). Sensitivity analyses yielded consistent results (Supplementary Figures S2, S3).

**Figure 3:**
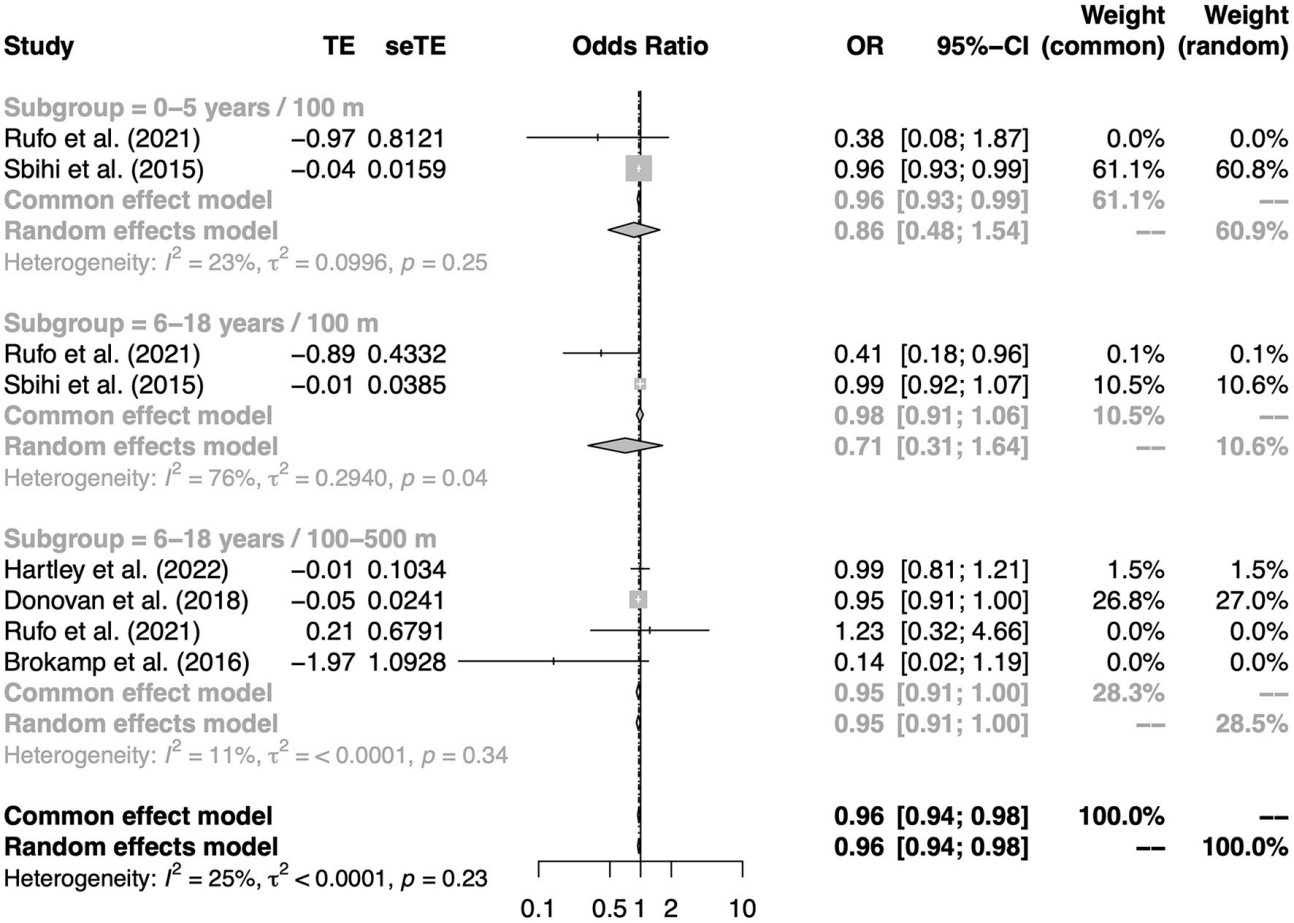
The association between residential greenness at birth and asthma in childhood. Subgroups were defined according to the age of the outcome measure and the buffers of the exposure measure.

Five birth cohort studies exploring the association between residential greenness exposure at birth and subsequent childhood asthma risk were considered for quantitative synthesis (17, 18, 26, 32, 40). The pooled results from these five studies showed a significant association (OR: 0.96, 95% CI: 0.94–0.98 per 0.1-unit change in NDVI, Figure 3), suggesting that living close to a greener environment at birth has a protective effect on the development of childhood asthma. Specifically, increased greenness within a buffer distance of 100–500 m around the residential address at birth might reduce the risk of asthma at 6–18 years of age (OR: 0.95, 95% CI: 0.91–1.00 per 0.1-unit change in NDVI, Supplementary Figure S3). To test the robustness of this result, sensitivity analyses were performed by one-by-one exclusion of studies with buffers of 100–500 m. The results of sensitivity analyses were consistent with the main results (Supplementary Figure S4). In addition, no significant publication bias was found for asthma outcomes (Supplementary Figures S5–S7).

Three studies investigated the association between greenness surrounding schools and the prevalence of asthma in children. Dzhambov et al. (35) reported that naturalness within 100 m of schools had no significant effect on asthma. However, Yu et al. (67) and Zeng et al. (68) reported that increased greenness within 100, 300, 500 and 1,000 m around schools was associated with a reduced risk of asthma.

In addition, several studies have evaluated the effects of greenness on adult asthma. Their findings are also contradictory. Using data from the Centers for Disease Control and Prevention 500 Cities Project, Donovan et al. (33) found that each 1 SD increase in NDVI was associated with a 3.8% increase in adult asthma rates. A study conducted in Norway and Sweden showed that lifelong exposure to greenness was not associated with asthma, but was a risk factor for poor lung function in adulthood (48). However, another study conducted among adults in southwestern Iran demonstrated that green space and home gardening were important protective factors against asthma (43).

### Association between greenness exposure and AR

**Figure 4:**
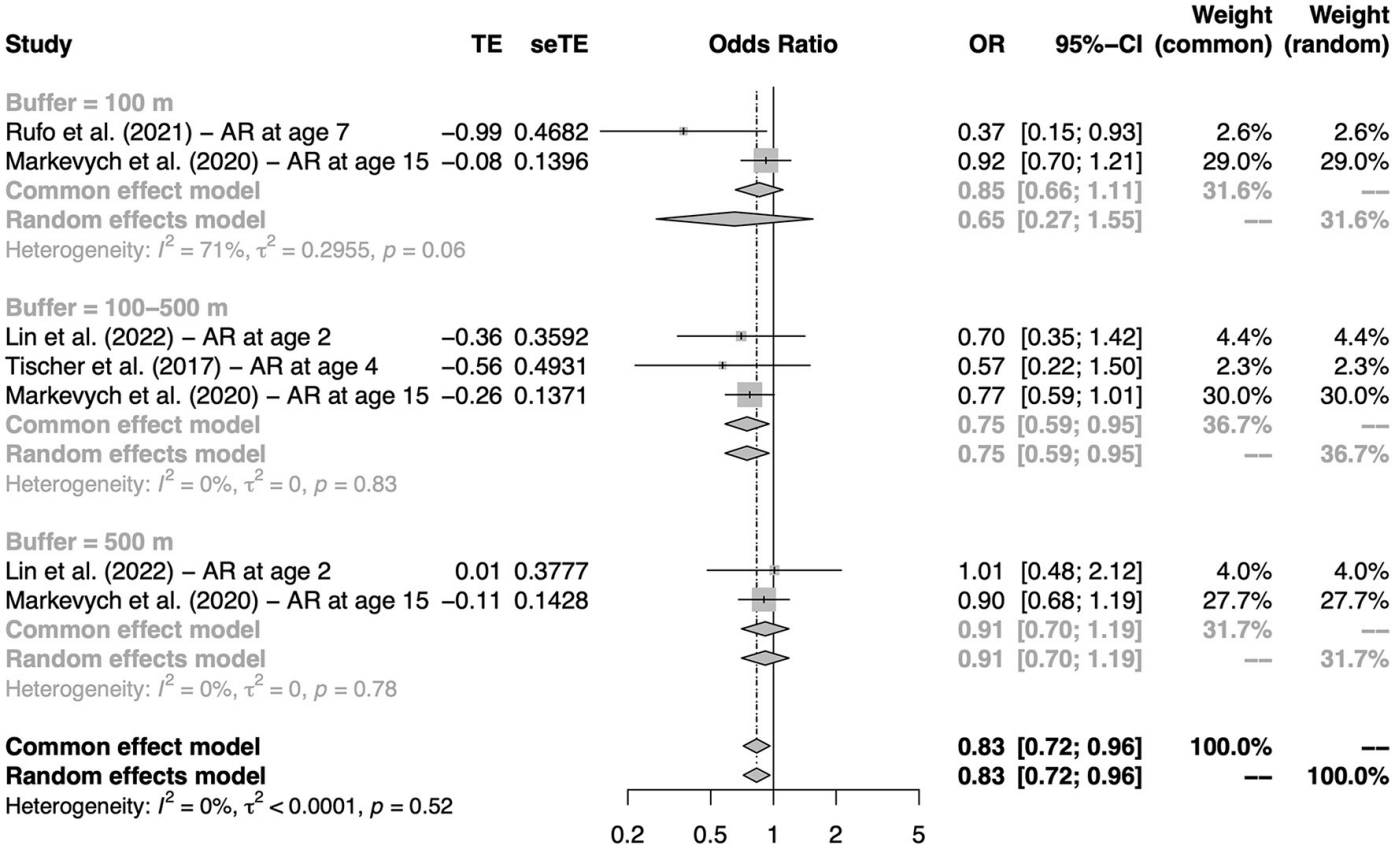
The association between residential greenness at birth and AR in childhood. Subgroups were defined according to the buffers of the exposure measure.

Four birth cohort studies reporting the association between residential NDVI at birth and the incidence of AR were considered for quantitative analysis (17, 54, 57, 64). As shown in Figure 4, the pooled OR for AR was 0.83 (95% CI: 0.72–0.96). Higher NDVI (3^rd^ tertile vs. 1^st^ tertile) was statistically significantly associated with a lower risk of AR within a buffer distance of 100–500 m (OR: 0.75, 95% CI: 0.59–0.95). No significant publication bias was found (Supplementary Figure S8).

Birth cohort studies by Fuertes et al. (16) and Gernes et al. (39) reported the effect of each 0.1 unit increase in NDVI on the incidence of AR. Fuertes et al. (16) reported that the direction of the association between NDVI and AR varied by region. Gernes et al. (39) reported that the association between NDVI or land cover-derived urban greenspace and AR was not statistically significant. In addition, although the number of studies is insufficient to be quantified, several cross-sectional studies have reported associations between greenness and AR. Dzhambov et al. (35) reported that among children aged 8–12 years, a higher distance from nature was associated with a higher prevalence of AR (OR: 1.32, 95% CI: 1.09–1.60), and conversely, the higher levels of NDVI and tree cover were slightly associated with a lower prevalence of AR. However, Li et al. (53) reported that no statistically significant associations were observed between residential NDVI values or distance to parks and AR among Chinese middle school students.

Studies conducted among Korean adults showed consistent results. Kim et al. (45) reported inverse associations between green areas (m^2^) and AR in Korean adults. Kwon et al. (49) reported that NDVI values were negatively correlated with the prevalence of AR.

### Association between greenness exposure and AD

There are not many studies examining the relationship between greenness and AD, and only qualitative analysis can be performed. Overall, studies have yielded inconsistent results. At present, it is difficult to clarify the effect of greenness exposure on atopic dermatitis.

Lin et al. reported that NDVI within 500 m of residence during pregnancy, 0–2 years of age, and the first 1,000 days of life was associated with a higher odd of having AD (54), whereas this association was not significant within a radius of 250 m (54). However, Lee et al. (50) reported the opposite finding. They reported that an increase in green space area within a buffer of 200 and 300 m around the home address was associated with a statistically significant decrease in AD risk (50). Another birth cohort study by Rufo et al. showed that high NDVI and species richness index (SRI) within 100 m at birth were not significantly associated with eczema at 4 or 7 years of age (17). Two cross-sectional studies conducted in school-aged children did not find a significant association between greenness and AD (35, 53). A cross-sectional study in Korean adults observed an inverse association between greenness and AD (45).

### Association between greenness exposure and food allergy

In general, studies exploring the effects of greenness exposure on food allergy have drawn inconsistent conclusions. Peters et al. reported that in Melbourne, Australia, increased residential greenness exposure in most buffer zones was associated with an increased risk of food allergy in 12-month-old infants, especially associated with peanut allergy and egg allergy (59). However, another birth cohort study conducted in Guangzhou, China, did not observe a significant association between greenness exposure and food allergy in children up to 2 years of age (54). A study of German birth cohort did not observe a significant association between residential greenness and food sensitization, but a positive relationship was found between allergenic trees and food sensitization (57).

### Association between greenness exposure and allergic rhinoconjunctivitis

Only a cross-sectional study conducted among 3,178 schoolchildren in Spain investigated the association between greenness exposure and allergic rhinoconjunctivitis, and no significant association was reported (28). In addition, a birth cohort study in young Chinese children collected data on allergic rhinoconjunctivitis, but no separate regression analysis was performed due to limited numbers in the preliminary analysis (54).

## Discussion

In this study, we systematically reviewed the association between greenness and various allergic diseases in adults and children. There was substantial heterogeneity among the available studies, including study populations, study designs, greenness measurement methods, spatial buffers, exposure time windows, definitions of allergic outcomes, adjustment for confounders, and scaling of effect estimates. These differences make it difficult to make comparisons across studies. Therefore, although 48 articles met our inclusion criteria, only five birth cohort studies, five cross-sectional studies, and one case-control study were included in the meta-analysis for asthma, and four birth cohort studies were included in the meta-analysis for AR. Our meta-analysis showed that exposure to more greenness at birth was associated with a reduced risk of asthma and AR during childhood. In addition, higher individual-level greenness exposure was associated with reduced odds of current asthma in children. Greenness in the 100–500 m buffer around residences appears to play an important protective role.

The atopic march begins with AD and food allergy in infancy and subsequently progresses to AR and asthma in childhood. Although the atopic march does not exactly follow a temporal pattern of genes and environment, it is interesting to assess the impact of environmental factors on allergic disease from a progression perspective. The available evidence is not yet sufficient to support an effect of greenness on AD, food allergy, and allergic rhinoconjunctivitis. Furthermore, although the present study showed a protective effect of greenness on AR and asthma, it is uncertain whether this result was obtained by chance or reflects a true pattern. Because there is substantial heterogeneity among existing studies and, more importantly, most studies did not specify greenness structure and allergens. We consider that it is important to identify vegetation types and allergens causing allergic diseases, because some vegetation can produce pollen allergens that directly affect allergic diseases, or there is cross-reactivity between some pollen and food allergens.

The results of our study are inconsistent with the meta-analysis of Lambert et al. (20). Lambert et al. reported a non-significant association between greenness and asthma and AR, with pooled ORs of 1.01 (95% CI: 0.93, 1.09) and 0.99 (95% CI: 0.87, 1.12), respectively (20). However, only three studies were included in the meta-analysis of asthma and two studies were included in the meta-analysis of AR. The limited number of studies may have masked the statistical significance. In addition, a meta-analysis conducted by Wu et al. (70) also reported that the effects of greenness exposure on asthma and AR were not significant. However, this study did not separately assess cross-sectional studies as well as birth cohorts in the meta-analysis, which may have masked the temporal relationship between exposure and outcome, leading to non-significant results. In this study, we found that greenness exposure at birth was a protective factor for asthma and AR in childhood. The differences between our study and others suggest that the effect of greenness exposure on allergic outcomes may be a long process, and that early life exposure may be a critical stage in influencing the development of allergic disease. Future studies should use study designs with higher levels of evidence to explore the effects of greenness exposure on allergic disease, as well as the atopic march.

Another important issue in existing studies is the method of exposure measurement. Most studies use NDVI to represent greenness exposure. NDVI can capture small-scale greenness in a standardized manner at multiple time points, with global availability and comparability. However, this approach may not be sufficiently detailed when assessing impacts on allergic diseases, because it does not allow for differentiation of specific vegetation types. Five studies have used LiDAR and obtained inconsistent conclusions (29, 31, 39, 46, 56). Compared to NDVI, LiDAR can provide higher resolution images and distinguish between different types of greenness, such as tree canopy, shrub, and grass cover (31). When evaluating the impact of greenness, it is important to consider the type and proportion of vegetation, especially in the field of allergic diseases. This is because different greenness structures can not only produce different types and concentrations of allergens, but also have different effects on air pollution and microbiota (31). However, LiDAR is not freely available to the public, which may be the reason why many studies do not use it. In this review, we did not perform a meta-analysis of studies using LiDAR due to the limited number of eligible studies. Future studies should explore the suitability of LiDAR for assessing the effects of greenness on allergic diseases. In addition, we recommend that future studies describe in detail the mean or median of each exposure level, as well as the number of participants and patients at each exposure level. This will help to further explore the linear or non-linear relationship between greenness and allergic diseases.

## Conclusions

Overall, our analysis supports that exposure to greener environments early in life may be a protective factor for AR and asthma in childhood. However, given the great heterogeneity among the included studies and the fact that most studies did not clarify the greenness structure and causative allergens, this result may only be applicable in some regions and populations. More high-quality studies are awaited to validate our results. In addition, most studies have focused on the terminal manifestations of the atopic march (AR and asthma), while only a few studies have explored the early manifestations of the atopic march (AD and food allergy). Future studies should continue to explore the effects of greenness on AD and food allergies.

## Data availability statement

The original contributions presented in the study are included in the article/Supplementary material, further inquiries can be directed to the corresponding author.

## Author contributions

XW: conceptualization, methodology, software, data curation, visualization, investigation, and writing—original draft preparation. NZ: data curation. YZ: conceptualization, methodology, and writing—reviewing and editing. All authors contributed to the article and approved the submitted version.

## References

[1] Sanchez-Borges M Martin BL Muraro AM Wood RA Agache IO Ansotegui IJ. The importance of allergic disease in public health: an iCAALL statement. World Allergy Organ J. (2018) 11:8. 10.1186/s40413-018-0187-2 29743965 PMC5921992

[2] Ober C Yao TC. The genetics of asthma and allergic disease: a 21st century perspective. Immunol Rev. (2011) 242:10–30. 10.1111/j.1600-065X.2011.01029.x 21682736 PMC3151648

[3] Burbank AJ Sood AK Kesic MJ Peden DB Hernandez ML. Environmental determinants of allergy and asthma in early life. J Allergy Clin Immunol. (2017) 140:1–12. 10.1016/j.jaci.2017.05.010 28673399 PMC5675123

[4] Platts-Mills TA. The allergy epidemics: 1870–2010. J Allergy Clin Immunol. (2015) 136:3–13. 10.1016/j.jaci.2015.03.048 26145982 PMC4617537

[5] Murrison LB Brandt EB Myers JB Hershey GKK. Environmental exposures and mechanisms in allergy and asthma development. J Clin Invest. (2019) 129:1504–15. 10.1172/JCI124612 30741719 PMC6436881

[6] Hartley K Ryan P Brokamp C Gillespie GL. Effect of greenness on asthma in children: a systematic review. Public Health Nurs. (2020) 37:453–60. 10.1111/phn.12701 31899558 PMC9292730

[7] Dewulf B Neutens T Van Dyck D De Bourdeaudhuij I Broekx S Beckx C. Associations between time spent in green areas and physical activity among late middle-aged adults. Geospat Health. (2016) 11:411. 10.4081/gh.2016.411 27903049

[8] Younan D Tuvblad C Li L Wu J Lurmann F Franklin M. Environmental determinants of aggression in adolescents: role of urban neighborhood greenspace. J Am Acad Child Adolesc Psychiatry. (2016) 55:591–601. 10.1016/j.jaac.2016.05.002 27343886 PMC4924128

[9] Hu CY Yang XJ Gui SY Ding K Huang K Fang Y. Residential greenness and birth outcomes: a systematic review and meta-analysis of observational studies. Environ Res. (2021) 193:110599. 10.1016/j.envres.2020.110599 33307084

[10] Liu XX Ma XL Huang WZ Luo YN He CJ Zhong XM. Green space and cardiovascular disease: a systematic review with meta-analysis. Environ Pollut. (2022) 301:118990. 10.1016/j.envpol.2022.118990 35181451

[11] Luo YN Huang WZ Liu XX Markevych I Bloom MS Zhao T. Greenspace with overweight and obesity: a systematic review and meta-analysis of epidemiological studies up to 2020. Obes Rev. (2020) 21:e13078. 10.1111/obr.13078 32677149

[12] Doubleday A Knott CJ Hazlehurst MF Bertoni AG Kaufman JD Hajat A. Neighborhood greenspace and risk of type 2 diabetes in a prospective cohort: the multi-ethncity study of atherosclerosis. Environ Health. (2022) 21:18. 10.1186/s12940-021-00824-w 35034636 PMC8762964

[13] Zare Sakhvidi MJ Yang J Siemiatycki J Dadvand P de Hoogh K Vienneau D. Greenspace exposure and cancer incidence: a 27-year follow-up of the French GAZEL cohort. Sci Total Environ. (2021) 787:147553. 10.1016/j.scitotenv.2021.147553 33989869

[14] Hanski I von Hertzen L Fyhrquist N Koskinen K Torppa K Laatikainen T. Environmental biodiversity, human microbiota, and allergy are interrelated. Proc Natl Acad Sci USA. (2012) 109:8334–9. 10.1073/pnas.1205624109 22566627 PMC3361383

[15] Fong KC Hart JE James P. A review of epidemiologic studies on greenness and health: updated literature through 2017. Curr Environ Health Rep. (2018) 5:77–87. 10.1007/s40572-018-0179-y 29392643 PMC5878143

[16] Fuertes E Markevych I Bowatte G Gruzieva O Gehring U Becker A. Residential greenness is differentially associated with childhood allergic rhinitis and aeroallergen sensitization in seven birth cohorts. Allergy Eur J Allergy Clin Immunol. (2016) 71:1461–71. 10.1111/all.12915 27087129

[17] Cavaleiro Rufo J Paciencia I Hoffimann E Moreira A Barros H Ribeiro AI. The neighbourhood natural environment is associated with asthma in children: a birth cohort study. Allergy. (2021) 76:348–58. 10.1111/all.14493 32654186

[18] Sbihi H Tamburic L Koehoorn M Brauer M. Greenness and incident childhood asthma: a 10-year follow-up in a population-based birth cohort. Am J Respir Crit Care Med. (2015) 192:1131–3. 10.1164/rccm.201504-0707LE 26517419

[19] Andrusaityte S Grazuleviciene R Kudzyte J Bernotiene A Dedele A Nieuwenhuijsen MJ. Associations between neighbourhood greenness and asthma in preschool children in Kaunas, Lithuania: a case-control study. BMJ Open. (2016) 6:e010341. 10.1136/bmjopen-2015-010341 27067890 PMC4838715

[20] Lambert KA Bowatte G Tham R Lodge C Prendergast L Heinrich J. Residential greenness and allergic respiratory diseases in children and adolescents—a systematic review and meta-analysis. Environ Res. (2017) 159:212–21. 10.1016/j.envres.2017.08.002 28803150

[21] Eze IC Hemkens LG Bucher HC Hoffmann B Schindler C Kunzli N. Association between ambient air pollution and diabetes mellitus in Europe and North America: systematic review and meta-analysis. Environ Health Perspect. (2015) 123:381–9. 10.1289/ehp.1307823 25625876 PMC4421762

[22] Wells GA Shea B O'Connell D Peterson J Welch V Losos M. The Newcastle-Ottawa Scale (NOS) for assessing the quality of nonrandomised studies in meta-analyses (2000).

[23] National Heart Lung and Blood Institute. Quality Assessment Tool for Observational Cohort and Cross-Sectional Studies. Bethesda, MD: National Heart, Lung, and Blood Institute (2014).

[24] Zhao J Li A Mei Y Zhou Q Li Y Li K. The association of arsenic exposure with hypertension and blood pressure: a systematic review and dose-response meta-analysis. Environ Pollut. (2021) 289:117914. 10.1016/j.envpol.2021.117914 34426185

[25] Alcock I White M Cherrie M Wheeler B Taylor J McInnes R. Land cover and air pollution are associated with asthma hospitalisations: a cross-sectional study. Environ Int. (2017) 109:29–41. 10.1016/j.envint.2017.08.009 28926750

[26] Brokamp C LeMasters GK Ryan PH. Residential mobility impacts exposure assessment and community socioeconomic characteristics in longitudinal epidemiology studies. J Expos Sci Environ Epidemiol. (2016) 26:428–34. 10.1038/jes.2016.10 26956935 PMC4913165

[27] Cilluffo G Ferrante G Fasola S Malizia V Montalbano L Ranzi A. Association between asthma control and exposure to greenness and other outdoor and indoor environmental factors: a longitudinal study on a cohort of asthmatic children. Int J Environ Res Public Health. (2022) 19:512. 10.3390/ijerph19010512 35010773 PMC8744738

[28] Dadvand P Villanueva CM Font-Ribera L Martinez D Basagaña X Belmonte J. Risks and benefits of green spaces for children: a cross-sectional study of associations with sedentary behavior, obesity, asthma, and allergy. Environ Health Perspect. (2015) 122:1329–35. 10.1289/ehp.1308038 25157960 PMC4256701

[29] De Roos AJ Kenyon CC Yen YT Moore K Melly S Hubbard RA. Does living near trees and other vegetation affect the contemporaneous odds of asthma exacerbation among pediatric asthma patients? J Urban Health Bull NY Acad Med. (2022) 99:533–48. 10.1007/s11524-022-00633-7 35467328 PMC9187838

[30] DePriest K Butz A Curriero FC Perrin N Gross D. Associations among neighborhood greenspace, neighborhood violence, and children's asthma control in an urban city. Ann Allergy Asthma Immunol. (2019) 123:608–10. 10.1016/j.anai.2019.10.003 31610235 PMC6915955

[31] Dong Y Liu H Zheng T. Association between green space structure and the prevalence of asthma: a case study of Toronto. Int J Environ Res Public Health. (2021) 18:5852. 10.3390/ijerph18115852 34072529 PMC8199317

[32] Donovan GH Gatziolis D Longley I Douwes J. Vegetation diversity protects against childhood asthma: results from a large New Zealand birth cohort. Nat Plants. (2018) 4:358. 10.1038/s41477-018-0151-8 29735984

[33] Donovan GH Landry SM Gatziolis D. The natural environment, plant diversity, and adult asthma: a retrospective observational study using the CDC's 500 Cities Project Data. Health Place. (2021) 67:102494. 10.1016/j.healthplace.2020.102494 33321458

[34] Douglas JA Archer RS Alexander SE. Ecological determinants of respiratory health: Examining associations between asthma emergency department visits, diesel particulate matter, and public parks and open space in Los Angeles, California. Prev Med Rep. (2019) 14:100855. 10.1016/j.pmedr.2019.100855 31024787 PMC6475663

[35] Dzhambov AM Lercher P Rudisser J Browning MHEM Markevych I. Allergic symptoms in association with naturalness, greenness, and greyness: a cross-sectional study in schoolchildren in the Alps. Environ Res. (2021) 198:110456. 10.1016/j.envres.2020.110456 33188758

[36] Eldeirawi K Kunzweiler C Zenk S Finn P Nyenhuis S Rosenberg N. Associations of urban greenness with asthma and respiratory symptoms in Mexican American children. Ann Allergy Asthma Immunol. (2019) 122:289–95. 10.1016/j.anai.2018.12.009 30557617

[37] Fuertes E Markevych I von Berg A Bauer C-P Berdel D Koletzko S. Greenness and allergies: evidence of differential associations in two areas in Germany. J Epidemiol Commun Health. (2014) 68:787–90. 10.1136/jech-2014-203903 24862831 PMC4112441

[38] Fuertes E Butland BK Ross Anderson H Carlsten C Strachan DP Brauer M. Childhood intermittent and persistent rhinitis prevalence and climate and vegetation: a global ecologic analysis. Ann Allergy Asthma Immunol. (2014) 113:386–392.e9. 10.1016/j.anai.2014.06.021 25065574

[39] Gernes R Brokamp C Rice GE Wright JM Kondo MC Michael YL. Using high-resolution residential greenspace measures in an urban environment to assess risks of allergy outcomes in children. Sci Total Environ. (2019) 668:760–7. 10.1016/j.scitotenv.2019.03.009 30865906 PMC6563346

[40] Hartley K Ryan PH Gillespie GL Perazzo J Wright JM Rice GE. Residential greenness, asthma, and lung function among children at high risk of allergic sensitization: a prospective cohort study. Environ Health. (2022) 21:1–12. 10.1186/s12940-022-00864-w 35549707 PMC9097404

[41] Hsieh CJ Yu PY Tai CJ Jan RH Wen TH Lin SW. Association between the first occurrence of asthma and residential greenness in children and teenagers in Taiwan. Int J Environ Res Public Health. (2019) 16:2076. 10.3390/ijerph16122076 31212779 PMC6616887

[42] Hu Y Chen Y Liu S Tan J Yu G Yan C. Residential greenspace and childhood asthma: an intra-city study. Sci Total Environ. (2023) 857:159792. 10.1016/j.scitotenv.2022.159792 36306842

[43] Idani E Raji H Maraghi E Aghababaeian H Madadizadeh F Dastoorpoor M. Risk factors associated with asthma among adults in Khuzestan, southwest Iran. Clin Epidemiol Global Health. (2020) 8:350–5. 10.1016/j.cegh.2019.09.001

[44] Ihlebæk C Aamodt G Aradi R Claussen B Thorén KH. Association between urban green space and self-reported lifestyle-related disorders in Oslo, Norway. Scand J Public Health. (2018) 46:589–96. 10.1177/1403494817730998 28976295

[45] Kim HJ Min JY Kim HJ Min KB. Association between green areas and allergic disease in Korean adults: a cross-sectional study. Ann Occup Environ Med. (2020) 32:e5. 10.35371/aoem.2020.32.e5 32082587 PMC7008584

[46] Kim D Ahn Y. The Contribution of neighborhood tree and greenspace to asthma emergency room visits: an application of advanced spatial data in Los Angeles County. Int J Environ Res Public Health. (2021) 18:347. 10.3390/ijerph18073487 33801701 PMC8036821

[47] Kuiper IN Markevych I Accordini S Bertelsen RJ Braback L Christensen JH. Associations of preconception exposure to air pollution and greenness with offspring asthma and hay fever. Int J Environ Res Public Health. (2020) 17:5828. 10.3390/ijerph17165828 32806543 PMC7459891

[48] Kuiper IN Svanes C Markevych I Accordini S Bertelsen RJ Braback L. Lifelong exposure to air pollution and greenness in relation to asthma, rhinitis and lung function in adulthood. Environ Int. (2021) 146:106219. 10.1016/j.envint.2020.106219 33126061

[49] Kwon MY Lee JS Park S. The effect of outdoor air pollutants and greenness on allergic rhinitis incidence rates: a cross-sectional study in Seoul, Korea. Int J Sustain Dev World Ecol. (2019) 26:258–67. 10.1080/13504509.2019.1570982

[50] Lee J-Y Lamichhane DK Lee M Ye S Kwon J-H Park M-S. Preventive effect of residential green space on infantile atopic dermatitis associated with prenatal air pollution exposure. Int J Environ Res Public Health. (2018) 15:102. 10.3390/ijerph15010102 29315266 PMC5800201

[51] Lee H-Y Wu Y-H Asri AK Chen T-H Pan W-C Yu C-P. Linkage between residential green spaces and allergic rhinitis among Asian children (case study: Taiwan). Landsc Urban Plan. (2020) 202:103868. 10.1016/j.landurbplan.2020.103868

[52] Lee S Baek J Kim SW Newman G. Tree canopy, pediatric asthma, and social vulnerability: an ecological study in Connecticut. Landsc Urban Planning. (2022) 225:104451. 10.1016/j.landurbplan.2022.104451

[53] Li L Hart JE Coull BA Cao S-J Spengler JD Adamkiewicz G. Effect of residential greenness and nearby parks on respiratory and allergic diseases among middle school adolescents in a Chinese city. Int J Environ Res Public Health. (2019) 16:991. 10.3390/ijerph16060991 30893887 PMC6466062

[54] Lin L Chen Y Wei J Wu S Wu S Jing J Dong G Cai L. The associations between residential greenness and allergic diseases in Chinese toddlers: a birth cohort study. Environ Res. (2022) 214:114003. 10.1016/j.envres.2022.114003 35931194

[55] Lovasi GS Quinn JW Neckerman KM Perzanowski MS Rundle A. Children living in areas with more street trees have lower prevalence of asthma. J Epidemiol Commun Health. (2008) 62:647–9. 10.1136/jech.2007.071894 18450765 PMC3415223

[56] Lovasi GS O'Neil-Dunne JP Lu JW Sheehan D Perzanowski MS Macfaden SW. Urban tree canopy and asthma, wheeze, rhinitis, and allergic sensitization to tree pollen in a New York City birth cohort. Environ Health Perspect. (2013) 121:494–500. 10.1289/ehp.1205513 23322788 PMC3620770

[57] Markevych I Ludwig R Baumbach C Standl M Heinrich J Herberth G. Residing near allergenic trees can increase risk of allergies later in life: LISA Leipzig study. Environ Res. (2020) 191:110132. 10.1016/j.envres.2020.110132 32853665

[58] Parmes E Pesce G Sabel CE Baldacci S Bono R Brescianini S. Influence of residential land cover on childhood allergic and respiratory symptoms and diseases: evidence from 9 European cohorts. Environ Res. (2020) 183:108953. 10.1016/j.envres.2019.108953 31818476

[59] Peters RL Sutherland D Dharmage SC Lowe AJ Perrett KP Tang MLK. The association between environmental greenness and the risk of food allergy: a population-based study in Melbourne, Australia. Pediatr Allergy Immunol. (2022) 33:e13749. 10.1111/pai.13749 35212044

[60] Pilat MA McFarland A Snelgrove A Collins K Waliczek TM Zajicek J. The effect of tree cover and vegetation on incidence of childhood asthma in Metropolitan statistical areas of Texas. Horttechnology. (2012) 22:631–7. 10.21273/HORTTECH.22.5.631

[61] Putra IGNE Astell-Burt T Feng X. Caregiver perceptions of neighbourhood green space quality, heavy traffic conditions, and asthma symptoms: group-based trajectory modelling and multilevel longitudinal analysis of 9,589 Australian children. Environ Res. (2022) 212:113187. 10.1016/j.envres.2022.113187 35358543

[62] Sbihi H Koehoorn M Tamburic L Brauer M. Asthma trajectories in a population-based birth cohort. Impacts of air pollution and greenness. Am J Respir Critic Care Med. (2017) 195:607–13. 10.1164/rccm.201601-0164OC 27606967

[63] Squillacioti G Bellisario V Levra S Piccioni P Bono R. Greenness availability and respiratory health in a population of urbanised children in North-Western Italy. Int J Environ Res Public Health. (2020) 17:108. 10.3390/ijerph17010108 31877852 PMC6981614

[64] Tischer C Gascon M Fernández-Somoano A Tardón A Materola AL Ibarluzea J. Urban green and grey space in relation to respiratory health in children. Eur Respir J. (2017) 49:1502112. 10.1183/13993003.02112-2015 28642307

[65] Winnicki MH Dunn RR Winther-Jensen M Jess T Allin KH Bruun HH. Does childhood exposure to biodiverse greenspace reduce the risk of developing asthma? Sci Total Environ. (2022) 850:157853. 10.1016/j.scitotenv.2022.157853 35940273

[66] Wu M Xie J Wang Y Tian Y. Greenness and eosinophilic asthma: findings from the UK Biobank. Eur Respir J. (2021) 58:2101597. 10.1183/13993003.01597-2021 34385276

[67] Yu H Zhou Y Wang R Qian Z Knibbs LD Jalaludin B. Associations between trees and grass presence with childhood asthma prevalence using deep learning image segmentation and a novel green view index. Environ Pollut. (2021) 286:117582. 10.1016/j.envpol.2021.117582 34438500

[68] Zeng XW Lowe AJ Lodge CJ Heinrich J Roponen M Jalava P. Greenness surrounding schools is associated with lower risk of asthma in schoolchildren. Environ Int. (2020) 143:105967. 10.1016/j.envint.2020.105967 32702595

[69] Rhew IC Vander Stoep A Kearney A Smith NL Dunbar MD. Validation of the normalized difference vegetation index as a measure of neighborhood greenness. Ann Epidemiol. (2011) 21:946–52. 10.1016/j.annepidem.2011.09.001 21982129 PMC3225119

[70] Wu B Guo X Liang M Sun C Gao J Xie P. Association of individual green space exposure with the incidence of asthma and allergic rhinitis: a systematic review and meta-analysis. Environ Sci Pollut Res Int. (2022) 2022:1–27. 10.1007/s11356-022-23718-x 36329245

