# Supplementary material for: Association between exposure to greenness and atopic march in children and adults—A systematic review and meta-analysis
Source: Front Public Health. 2023 Jan 9;10:1097486. doi: 10.3389/fpubh.2022.1097486 (PMC9868616; doi:10.3389/fpubh.2022.1097486)
Supplement: Supplementary file 1 [file Data_Sheet_1.docx]

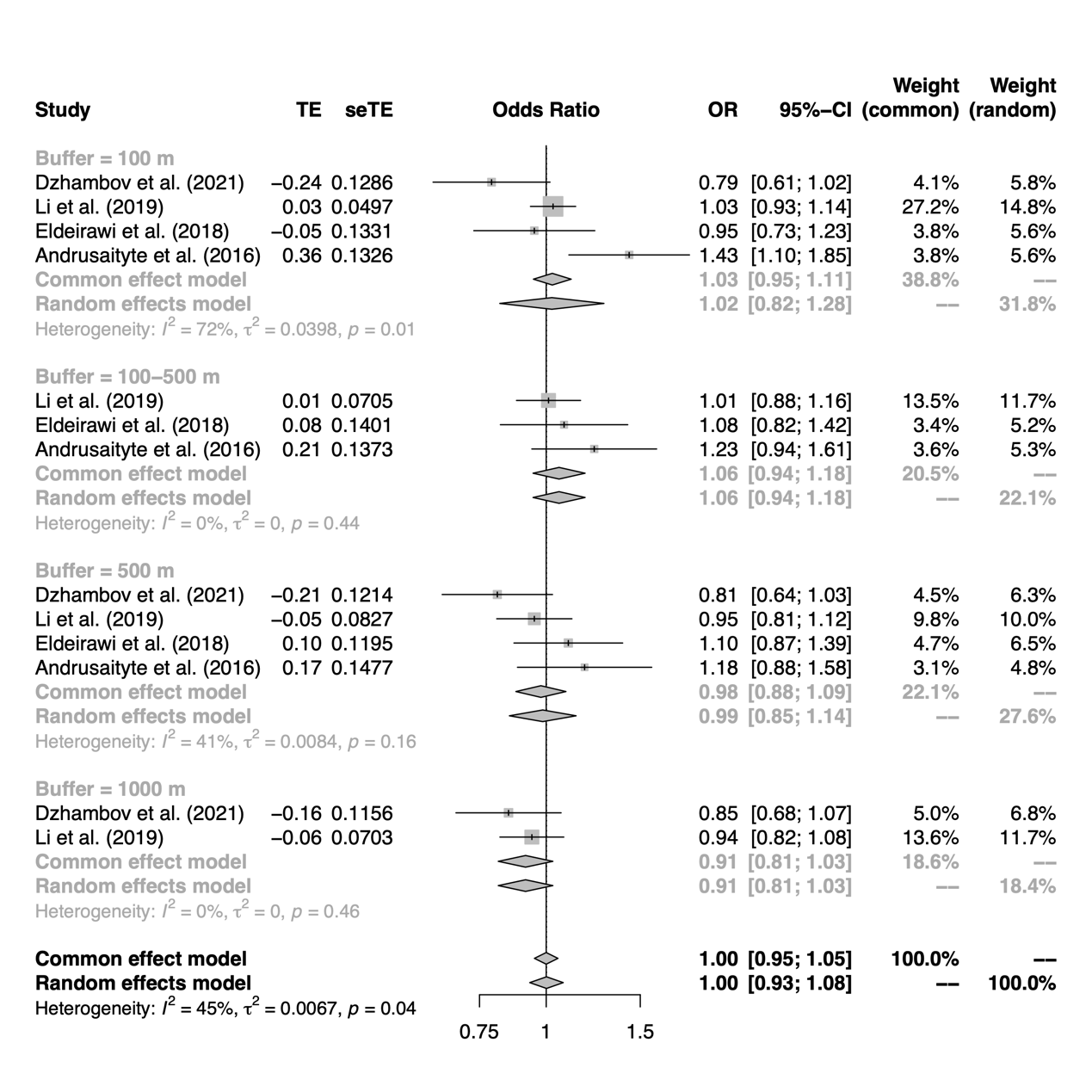
**Figure S1.** The association between residential greenness and ever asthma


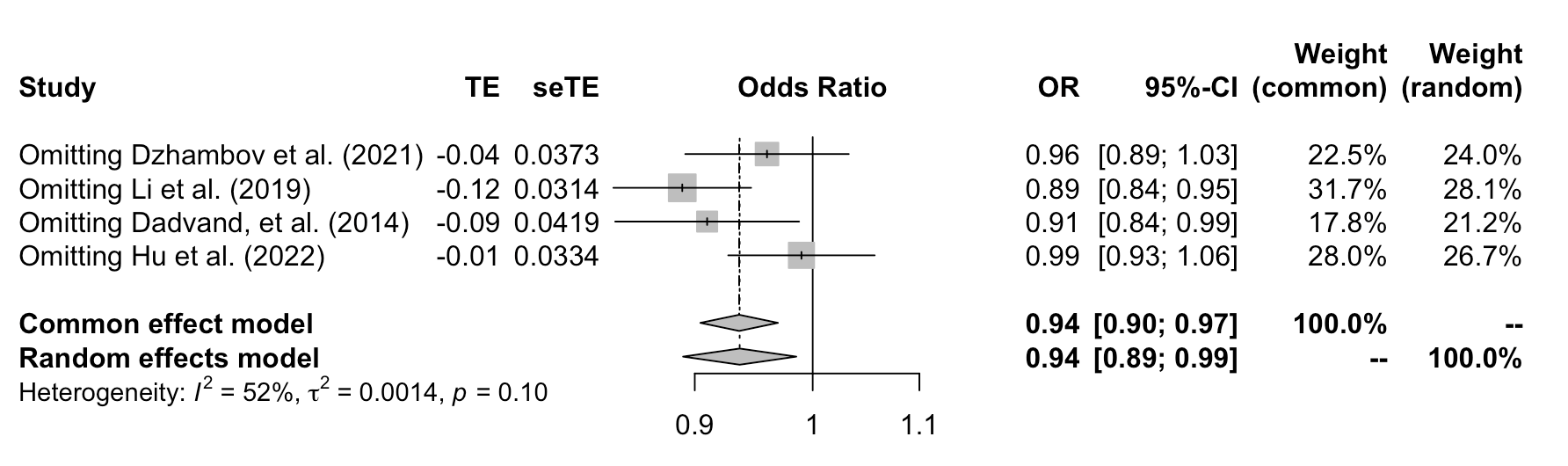


**Figure S2.** The association between residential greenness and current asthma in childhood after sensitivity analysis.


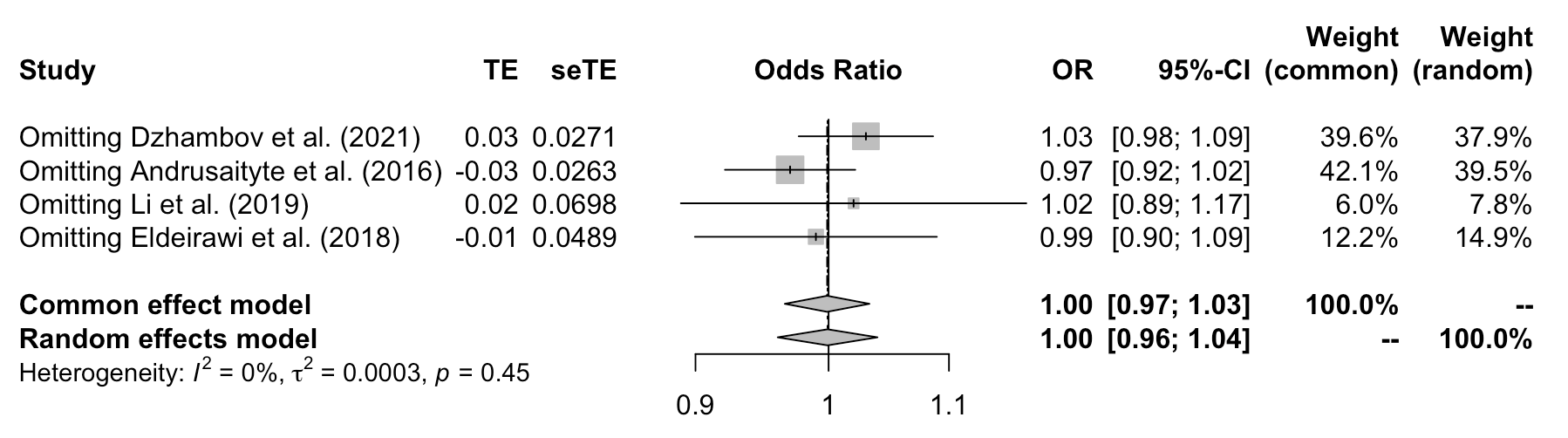


**Figure S3.** The association between residential greenness and ever asthma in childhood after sensitivity analysis.


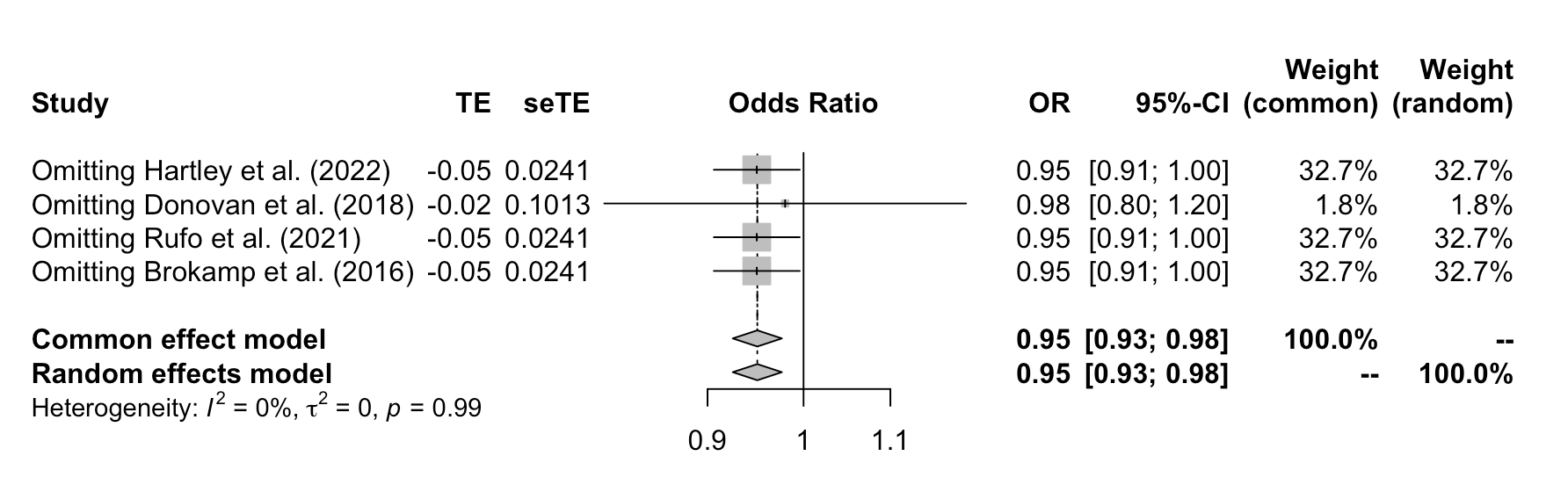


**Figure S4.** The association between residential greenness at birth and asthma in childhood after sensitivity analysis.


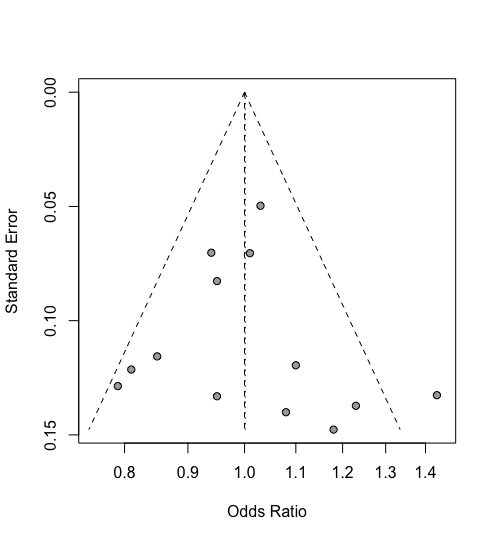


**Figure S5.** Funnel plot for the association between residential greenness and ever asthma. P-value of Begg's Test: 0.7630, P-value of Egger's test: 0.3930


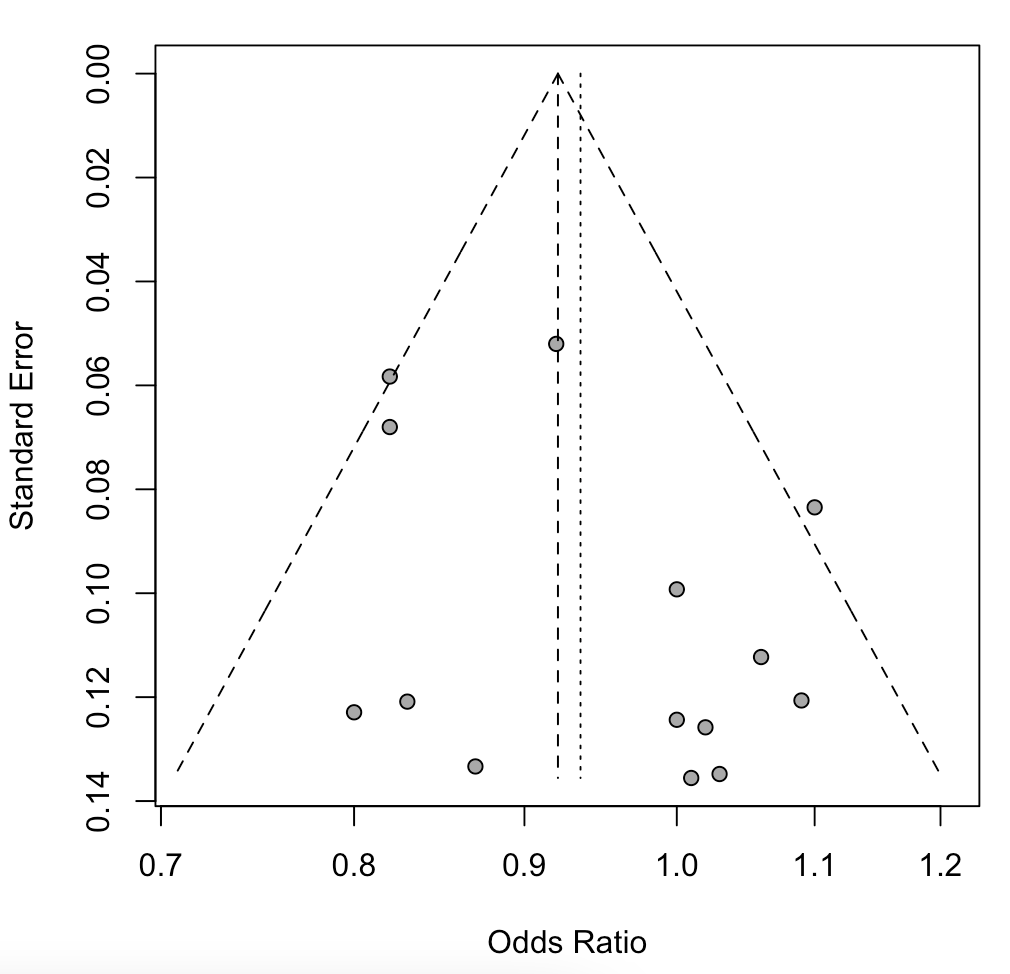


**Figure S6.** Funnel plot for the association between residential greenness and current asthma. P-value of Begg's Test: 0.1333, P-value of Egger's test: 0.6222


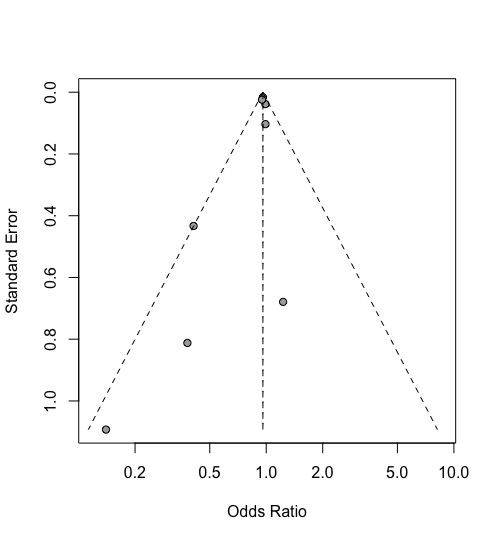


**Figure S7.** Funnel plot for the association between residential greenness at birth and asthma in childhood. P-value of Begg's Test: 0.1353, P-value of Egger's test: 0.3223


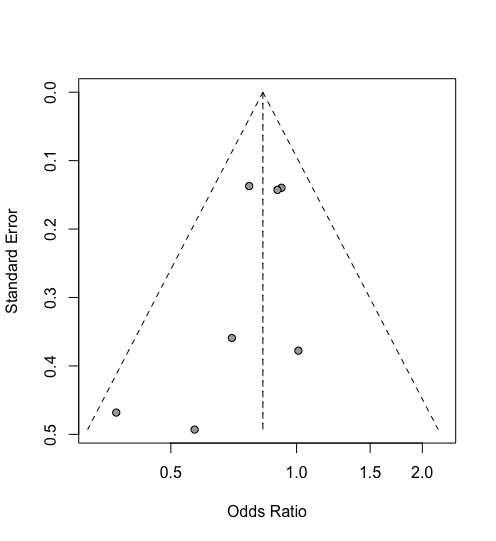


**Figure S8.** Funnel plot for the association between residential greenness at birth and AR in childhood. P-value of Begg's Test: 0.1563, P-value of Egger's test: 0.1765

**Table S1.** PRISMA 2020 Checklist

| **Section and Topic** | **Item #** | **Checklist item** | **Location where item is reported** |
| --- | --- | --- | --- |
| **TITLE** | | |  |
| Title | 1 | Identify the report as a systematic review. | Page 1 of the manuscript |
| **ABSTRACT** | | |  |
| Abstract | 2 | See the PRISMA 2020 for Abstracts checklist (Provide an explicit statement of the main objective(s) or question(s) the review addresses). | Page 2 of the manuscript |
| **INTRODUCTION** | | |  |
| Rationale | 3 | Describe the rationale for the review in the context of existing knowledge. | Page 4 of the manuscript |
| Objectives | 4 | Provide an explicit statement of the objective(s) or question(s) the review addresses. | Page 5 of the manuscript |
| **METHODS** | | |  |
| Eligibility criteria | 5 | Specify the inclusion and exclusion criteria for the review and how studies were grouped for the syntheses. | Pages 5 and 6 of the manuscript |
| Information sources | 6 | Specify all databases, registers, websites, organisations, reference lists and other sources searched or consulted to identify studies. Specify the date when each source was last searched or consulted. | Page 5 of the manuscript |
| Search strategy | 7 | Present the full search strategies for all databases, registers and websites, including any filters and limits used. | Page 5 of the manuscript, and Table S2 |
| Selection process | 8 | Specify the methods used to decide whether a study met the inclusion criteria of the review, including how many reviewers screened each record and each report retrieved, whether they worked independently, and if applicable, details of automation tools used in the process. | Pages 6, 7, and 8 of the manuscript |
| Data collection process | 9 | Specify the methods used to collect data from reports, including how many reviewers collected data from each report, whether they worked independently, any processes for obtaining or confirming data from study investigators, and if applicable, details of automation tools used in the process. | Page 6 of the manuscript |
| Data items | 10a | List and define all outcomes for which data were sought. Specify whether all results that were compatible with each outcome domain in each study were sought (e.g. for all measures, time points, analyses), and if not, the methods used to decide which results to collect. | Page 6 of the manuscript, Table 1, and Table S3 |
|  | 10b | List and define all other variables for which data were sought (e.g. participant and intervention characteristics, funding sources). Describe any assumptions made about any missing or unclear information. | Table S3 |
| Study risk of bias assessment | 11 | Specify the methods used to assess risk of bias in the included studies, including details of the tool(s) used, how many reviewers assessed each study and whether they worked independently, and if applicable, details of automation tools used in the process. | Page 7 of the manuscript |
| Effect measures | 12 | Specify for each outcome the effect measure(s) (e.g. risk ratio, mean difference) used in the synthesis or presentation of results. | Page 8 of the manuscript |
| Synthesis methods | 13a | Describe the processes used to decide which studies were eligible for each synthesis (e.g. tabulating the study intervention characteristics and comparing against the planned groups for each synthesis (item #5)). | Pages 7 and 8 of the manuscript |
|  | 13b | Describe any methods required to prepare the data for presentation or synthesis, such as handling of missing summary statistics, or data conversions. | Page 7 of the manuscript |
|  | 13c | Describe any methods used to tabulate or visually display results of individual studies and syntheses. | Page 8 of the manuscript |
|  | 13d | Describe any methods used to synthesize results and provide a rationale for the choice(s). If meta-analysis was performed, describe the model(s), method(s) to identify the presence and extent of statistical heterogeneity, and software package(s) used. | Pages 8 and 9 of the manuscript |
|  | 13e | Describe any methods used to explore possible causes of heterogeneity among study results (e.g. subgroup analysis, meta-regression). | Page 8 of the manuscript |
|  | 13f | Describe any sensitivity analyses conducted to assess robustness of the synthesized results. | Page 13 of the manuscript |
| Reporting bias assessment | 14 | Describe any methods used to assess risk of bias due to missing results in a synthesis (arising from reporting biases). | Page 8 of the manuscript |
| Certainty assessment | 15 | Describe any methods used to assess certainty (or confidence) in the body of evidence for an outcome. | Page 7 of the manuscript |
| **RESULTS** | | |  |
| Study selection | 16a | Describe the results of the search and selection process, from the number of records identified in the search to the number of studies included in the review, ideally using a flow diagram. | Figure 1 |
|  | 16b | Cite studies that might appear to meet the inclusion criteria, but which were excluded, and explain why they were excluded. | Figure 1 |
| Study characteristics | 17 | Cite each included study and present its characteristics. | Table 1 and Table S3 |
| Risk of bias in studies | 18 | Present assessments of risk of bias for each included study. | Figure S5 to Figure S8 |
| Results of individual studies | 19 | For all outcomes, present, for each study: (a) summary statistics for each group (where appropriate) and (b) an effect estimate and its precision (e.g. confidence/credible interval), ideally using structured tables or plots. | Table S3 |
| Results of syntheses | 20a | For each synthesis, briefly summarise the characteristics and risk of bias among contributing studies. | Table S3, pages 10 and 11 of the manuscript |
|  | 20b | Present results of all statistical syntheses conducted. If meta-analysis was done, present for each the summary estimate and its precision (e.g. confidence/credible interval) and measures of statistical heterogeneity. If comparing groups, describe the direction of the effect. | Figure 2, Figure 3, Figure 4, and Figure S1; Pages 12, 13, and 14 of the manuscript |
|  | 20c | Present results of all investigations of possible causes of heterogeneity among study results. | Pages 10, and 11 of the manuscript |
|  | 20d | Present results of all sensitivity analyses conducted to assess the robustness of the synthesized results. | Figure S2, S3, and S4 |
| Reporting biases | 21 | Present assessments of risk of bias due to missing results (arising from reporting biases) for each synthesis assessed. | Page 11 of the manuscript |
| Certainty of evidence | 22 | Present assessments of certainty (or confidence) in the body of evidence for each outcome assessed. | Table S4 to Table S6 |
| **DISCUSSION** | | |  |
| Discussion | 23a | Provide a general interpretation of the results in the context of other evidence. | Page 16 of the manuscript |
|  | 23b | Discuss any limitations of the evidence included in the review. | Page 18 of the manuscript |
|  | 23c | Discuss any limitations of the review processes used. | Page 18 of the manuscript |
|  | 23d | Discuss implications of the results for practice, policy, and future research. | Page 18 of the manuscript |
| **OTHER INFORMATION** | | |  |
| Registration and protocol | 24a | Provide registration information for the review, including register name and registration number, or state that the review was not registered. | The review was not registered |
|  | 24b | Indicate where the review protocol can be accessed, or state that a protocol was not prepared. | Review protocol was not prepared |
|  | 24c | Describe and explain any amendments to information provided at registration or in the protocol. | The review was not registered |
| Support | 25 | Describe sources of financial or non-financial support for the review, and the role of the funders or sponsors in the review. | Page 19 of the manuscript |
| Competing interests | 26 | Declare any competing interests of review authors. | Page 19 of the manuscript |
| Availability of data, code and other materials | 27 | Report which of the following are publicly available and where they can be found: template data collection forms; data extracted from included studies; data used for all analyses; analytic code; any other materials used in the review. | Page 19 of the manuscript |

**Table S2.** Search strings in each database.

| **Database** | **Search strings** |
| --- | --- |
| PubMed | ("greenspace" or "green space" or "greenness" or "greenery" or "grassland" or "grass land" or "green land" or "grass cover" or "natural area" or "vegetation") and ("allergy" or "allergic disease" or "asthma" or "hay fever" or "allergic rhinitis" or "eczema" or "atopic dermatitis" or "allergic respiratory diseases") |
| ISI Web of Science | ("greenspace" or "green space" or "greenness" or "greenery" or "grassland" or "grass land" or "green land" or "grass cover" or "natural area" or "vegetation") and ("allergy" or "allergic disease" or "asthma" or "hay fever" or "allergic rhinitis" or "eczema" or "atopic dermatitis" or "allergic respiratory diseases") |
| EMBASE | ('greenspace'/exp OR 'greenspace' OR 'green space'/exp OR 'green space' OR 'greenness'/exp OR 'greenness' OR 'greenery' OR 'grassland'/exp OR 'grassland' OR 'grass land' OR 'green land' OR 'grass cover' OR 'natural area' OR 'vegetation'/exp OR 'vegetation') AND ('allergy'/exp OR 'allergy' OR 'allergic disease'/exp OR 'allergic disease' OR 'asthma'/exp OR 'asthma' OR 'hay fever'/exp OR 'hay fever' OR 'allergic rhinitis'/exp OR 'allergic rhinitis' OR 'eczema'/exp OR 'eczema' OR 'atopic dermatitis'/exp OR 'atopic dermatitis' OR 'allergic respiratory diseases') |
| Scopus | ("greenspace" or "green space" or "greenness" or "greenery" or "grassland" or "grass land" or "green land" or "grass cover" or "natural area" or "vegetation") and ("allergy" or "allergic disease" or "asthma" or "hay fever" or "allergic rhinitis" or "eczema" or "atopic dermatitis" or "allergic respiratory diseases") |

**Table S3.** The exposure definitions, outcome definitions, statistical models, estimates, and adjusted confounders in each study.

| Authors (Year) | Exposure definition | Outcome definition | Statistical model | Estimate (95% CI) | Confounders |  |
| --- | --- | --- | --- | --- | --- | --- |
|  |  |  |  |  |  |  |
| Alcock et al. (2017) | 1) Lower super output area (LSOA) percentage of green space 2) LSOA percentage of gardens 3) the density of mature trees, or closely grouped tree crowns where clustered canopies were at the same height | Asthma hospitalizations | Negative binomial regression models | Per SD increase in vegetation exposure on asthma rate: Greenspace: -124.64 (-141.84, -107.46) Gardens: -93.35 (-111.29, -75.41) Trees: -66.8 (− 78.66, − 54.94) | Deprivation component variables and government office region |  |
| Andrusaityte et al. (2016) | Residential surrounding greenness (mean NDVI at 100, 300, and 500 m areas of exact residential address); Distance to a city park (>1000 m or ≤1000 m) | Responses to the standardised ISAAC questionnaire completed by parents were used to identify children with asthma. Childhood asthma was identified by an affirmative response to the question: ‘Has you child ever had doctor-diagnosed asthma?’ | Logistic regression model | Per IQR increase in NDVI (OR): 100 m: 1.43 (1.10, 1.85) 300 m: 1.23 (0.94, 1.61) 500 m: 1.18 (0.88, 1.57) For above median NDVI (OR): 100 m: 1.19 (0.79, 1.79) 300 m: 1.17 (0.78, 1.76) 500 m: 1.39 (0.92, 2.10) For distance to a city park (OR): >1000 m: 0.96 (0.55, 1.68) | Parental asthma, maternal education, age at childbirth, smoking during pregnancy, breastfeeding, antibiotic use during the first year of life, keeping a cat during the past 12 months, living in a flat, time spent in green space. |  |
| Brokamp et al. (2016) | NDVI at 400 m around home address from a single image in June 2000, for each address recorded over the first seven years of life. | Asthma at age 7, recorded by a combination of self-reported symptoms of asthma and had either bronchial hyper-reactivity or a positive MCCT (PC20 ≤4 mg/ml methacholine concentration) | Logistic regression model | NDVI at birth address: OR: 0.14 (0.02, 1.19) NDVI at 7 yr address: OR: 0.18 (0.02, 1.70) Mean NDVI over all addresses: 0.15 (0.01, 2.04) | - |  |
| Cilluffo et al. (2022) | NDVI was achieved for each house location involved in the study at an approximate resolution of 200 square meters and was evaluated at each time visit. | Asthma control was assessed using the Childhood Asthma Control Test (C-ACT). Uncontrolled asthma (UA) was defined as C-ACT or ACT total score ≤ 19; controlled asthma (C) was defined as C-ACT or ACT total score > 19. | Logistic regression model | NDVI ≤ 0.21 (OR): 2.662 (1.043, 6.799) | Comorbidity (Y vs. N); persistent asthma (Y vs. N, time varying); atopy (Y vs. N); parental education <8 years (Y vs. N) |  |
| Dadvand, et al. (2014) | Residential surrounding greenness (mean NDVI in buffers of 100 m, 250 m, 500 m, and 1000 m around her/his geocoded address of residence); Residential proximity to green spaces (two binary variables indicating whether the child’s residential address was located within 300 m separately from a park or forest). | Current asthma and current allergic rhinoconjunctivitis was assessed using the validated International Study of Asthma and Allergies in Childhood (ISAAC) questionnaire. | Logistic mixed-effects models | Current asthma / Current allergic rhinoconjunctivitis: Per IQR increase in NDVI (OR): 100 m: 1.00 (0.82, 1.21) / 0.97 (0.88, 1.08) 250 m: 1.00 (0.78, 1.27) / 0.98 (0.87, 1.12) 500 m: 1.03 (0.79, 1.34) / 1.03 (0.90, 1.18) 1000 m: 1.06 (0.85, 1.32) / 1.05 (0.94, 1.18) Living within 300 m of parks (OR): 1.60 (1.09, 2.36) / 1.10 (0.90, 1.35) Living within 300 m of forests (OR): 1.02 (0.56, 1.87) / 1.27 (0.94, 1.70) | Child’s sex and age, exposure to environmental tobacco smoke at home, having older siblings, type of school (public vs. private), parental education, and parental history of asthma. |  |
| De Roos et al. (2022) | Overall greenness: median NDVI within a 250-m buffer; Canopy and grass/shrub cover: data from Light Detection and Ranging (LiDAR) data and orthoimagery were used to calculate the percentage of land area covered by tree canopy within a 250-m buffer, and separately for low vegetation, scrub/shrub, and emergent wetlands (shortened as “grass/shrub”). | An asthma exacerbation case was defined as a face-to-face physician visit in a Children’s Hospital of Philadelphia facility with a diagnosis code for asthma and a linked prescription of systemic steroid. | Generalized estimating equations | Per IQR (0.16) increase in NDVI-250 m (OR): 0.98 (0.93,1.03) Per IQR (10.2%) increase in tree canopy cover (OR): 0.98 (0.95, 1.02) Per IQR (6.1%) increase in grass/shrub cover (OR): 0.99 (0.96–1.02) | Calendar month, day-of-week, census tract population density, and census tract SES index |  |
| DePriest et al. (2020) | Greenspace (NDVI calculated using satellite data) | Level of asthma control, categorized as not well controlled (NWC) or very poorly controlled (VPC), was based on parents’ report of their child’s asthma symptom days, symptom nights, activity limitation, and rescue inhaler use. | Logistic regression model | OR: 0.98 (0.79, 1.21) |  |  |
| Dong et al. (2021) | The data of the vegetation types (trees, shrubs, and grass) were derived from multispectral satellite imagery with the assistance of LiDAR information. | The prevalence of asthma |  | At all ages: coefficients = -0.19, p > 0.05 At 0–19 years: coefficients = -0.27, p ≤ 0.01 At 20+ years: coefficients = -0.13, p > 0.05 | Greenness (measured by the percentage of green space), income, number of family members, and ethnic group. |  |
| Donovan et al. (2018) | Greenness index: mean NDVI for each meshblock for each year from 1998 to 2016 (the mean area of an urban meshblock is equal to the area of a circle with a 255 m radius); Exposures were calculated for three different periods of a child’s life: prenatal to age 18, prenatal to age 2 and age 2+ to age 18. | The prevalence of asthma. Asthma was defined on the basis of two criteria: (1) a diagnosis of asthma in hospital (ICD-10 codes J45 or J46); or (2) having received seven or more prescriptions for inhaled corticosteroids or an inhaled beta-adrenoceptor agonist between 2005 and 2016. | Logistic regression model | Per 1 SD increase in mean lifetime NDVI (OR): 0.940 (0.901, 0.981) | Roads, air pollution, ethnicity, gender, birth outcomes, parents’ occupation, parents’ education, parents’ smoking status, antibiotic use, number of siblings, meshblock size, birth order,the diversity of a child’s greenness exposure, and child’s exposure to specific land-cover types. |  |
| Donovan et al. (2021) | 1) Exposure to plant diversity: plant-occurrence data from the Global Biodiversity Information Facility (GBIF) were used to calculate plant-diversity metrics;  2) Exposure to overall greenness: average of the 250 m NDVI pixels within each tract boundary. | The rate of adult asthma | Mixed linear model | Maximum NDVI (coefficient):  0.0383 (0.0290,0.0475) Taxonomic diversity (coefficient): -0.0528 (-0.0638, -0.0418) | Race, ethnicity, socioeconomic status, behavioral risk factors, health prevention, air pollution, and overall greenness |  |
| Douglas et al. (2019) | Acres of PPOS across 2347 census tracts in Los Angeles, California | Asthma emergency department visits | Ordinary least squares (OLS) regression model | Estimate: -8.05, p<0.001 | Percentage of residents living two times below the federal poverty line, percentage of residents under the age of 10, and number of African American/Black, Latina/o, White, Asian, American Indian/Alaska Native, Native Hawaiian/Pacific Islander, and Other residents per square mile. |  |
| Dzhambov et al. (2021) | Residential greenness (NDVI, tree cover, agricultural cover, and domestic garden with 500-m buffers); School exposure (D2N, NDVI, tree cover, and agricultural cover with 100-m buffers). | Current asthma symptoms, ever asthma symptoms, ever AR, ever eczema symptoms. | Logistic regression model | Per IQR increase in school greenness associated with ever asthma/AR/eczema (OR): NDVI 100-m: 0.99 (0.85, 1.15) / 0.86 (0.74, 0.99) / 0.96 (0.83, 1.12) Per IQR increase in residential greenness associated with ever asthma/AR/eczema (OR): NDVI 500-m: 0.81 (0.64, 1.03) / 0.83 (0.67, 1.03) / 0.95 (0.75, 1.21) | Age, gender, maternal education, low birth weight, maternal smoking during pregnancy, duration of breastfeeding, cumulative risk of secondhand smoking/pneumonia/bronchitis in the 1st year of life, number of green months during pregnancy, geographic region. |  |
| Eldeirawi et al. (2018) | NDVI based on satellite imagery within buffers of 100, 250, and 500 m of each child’s residence. | Lifetime asthma: lifetime physician-diagnosed asthma | Multilevel mixed-effect logistic regression | Per IQR increase in NDVI (OR): 100 m: 0.95 (0.73, 1.23) 250 m: 1.08 (0.82, 1.42) 500 m: 1.10 (0.87, 1.39) | age, sex, country of birth, place child was born or lived during first year of life, family history of asthma or allergies, number of siblings, child attended preschool or day care, access to a regular physician or clinic, child had an ear infection during first year of life, child had a viral infection during first year of life, child took antibiotics during first year of life, child ever breastfed, current exposure to cats and/or dogs, smoker present in the home at the time of child’s birth, current smoker present in the home, proximity to traffic, population density, neighborhood deprivation, percentage of residents who identify as Mexican, and number of total crimes reported in 2004. |  |
| Fuertes et al. (2014) | Residential greenness (mean NDVI in circular 500, 800, 1000 and 3000 m buffers around the birth, 6- and 10-year participant addresses) | AR was evaluated annually from age 3 to 10 years. | Generalized estimation equations | For AR during the first 10 years of life (OR): Per IQR increase in NDVI-500 m: 1.03 (0.89 to 1.19) For eyes and nose symptoms during the first 10 years of life (OR): Per IQR increase in NDVI-500 m: 1.00 (0.88 to 1.14) | Age, sex, parental history of atopy, older siblings, maternal smoking during pregnancy, tobacco smoke exposure in the home (1–4 years), parental education, cohort and geographical area |  |
| Fuertes et al. (2014) | NDVI values at the central-level and country-level were calculated. Mean, maximum, minimum, SD and maximum variance were calculated. | Prevalence of intermittent (at least 1 symptom report but not for 2 consecutive months) and persistent (symptoms for at least 2 consecutive months) rhinitis symptoms. | Linear regression mixed models | Association of a 1-unit increase in central-level NDVI with intermittent rhinitis / persistent rhinitis prevalence: Mean: −9.05 (−19.66 to 1.55) / 8.37 (−1.04 to 17.77) | Gross national income (GNI) per capita, population density, climate type, country as a random intercept and fixed effects for both the center- and country-level representation of each explanatory variable, except for GNI per capita. |  |
| Fuertes et al. (2016) | Residential greenness (mean NDVI within a 500 m and 1000 m circular buffer, which was derived from satellite maps at the time of birth of the participants) | AR was defined based on a diagnosis during a physician assessment at a follow-up visit in CAPPS and SAGE, parental report of a doctor’s diagnosis in GINIplus and LISAplus, parental symptom report in PIAMA and BAMSE, and parental symptom or treatment report in MACS. | Logistic regression model | For childhood (6–8 years) AR (OR): Per 0.2 increase in NDVI: 500 m: 1.00 (0.69, 1.45) Per IQR increase in NDVI: 500 m: 1.00 (0.80, 1.25) For early adolescence (10–12 years) AR (OR): Per 0.2 increase in NDVI: 500 m: 0.96 (0.71, 1.30) Per IQR increase in NDVI: 500 m: 0.97 (0.82, 1.16) | Sex, age, parental atopy, older siblings, maternal smoking during pregnancy, secondhand smoke exposure, socioeconomic status, group, region, and cohort. |  |
| Gernes et al. (2019) | NDVI and land cover-derived UGS within a radial buffer of 400 m around participants’ primary home addresses. Cloud-free in-leaf imagery collected on June 19, 2010 were used. | AR was assessed at ages 1, 2, 3, 4, and 7 years. AR was defined as having allergic sensitization (to any of the 15 aeroallergens tested) and a positive parental/guardian response to the question: “in the past 12 months, has your child ever had a problem with sneezing, or a runny, or a blocked nose when he/she DID NOT have a cold/flu?” | Logistic regression model | For AR at age 7 (OR):  Per IQR increase in NDVI-100 m: 0.93 (0.74, 1.17) Per IQR increase in NDVI-400 m: 0.95 (0.76, 1.20) Per IQR increase in NDVI-800 m: 0.92 (0.72, 1.16) Per 10% increase in UGS-100 m: 0.93 (0.80, 1.07) Per 10% increase in UGS-400 m: 0.90 (0.69, 1.19) Per 10% increase in UGS-800 m: 0.87 (0.73, 1.05) For AR at age 4 (OR):  Per IQR increase in NDVI-100 m: 1.08 (0.80, 1.46) Per IQR increase in NDVI-400 m: 0.86 (0.60, 1.24) Per IQR increase in NDVI-800 m: 0.78 (0.55, 1.11) | Race, sex, environmental tobacco smoke exposure, exposure to traffic-related air pollution, mother’s education (7 years), and neighborhood SES (7 years) |  |
| Hartley et al. (2022) | Residential greenness (NDVI within 200, 400, and 800 m distances from geocoded home addresses at birth, age 7 years, and across childhood) | Asthma was assessed at age 7 years, defined as experiencing asthma symptoms during the study visit and exhibiting bronchodilation (≥12% increase in FEV1 after nebulized leucovorin) or bronchoprovocation (≥20% decrease in baseline FEV1 after inhaled methacholine). | Logistic regression model | Relationship between per 0.1-unit change in NDVI and asthma across different time windows (OR): At birth: 200 m: 0.99 (0.8, 1.2) 400 m: 0.99 (0.8, 1.3) 800 m: 0.84 (0.6, 1.1) Age 7: 200 m: 0.94 (0.7, 1.2) 400 m: 1.01 (0.8, 1.3) 800 m: 1.01 (0.8, 1.4) Across Childhood: 200 m: 0.97 (0.7, 1.3) 400 m: 1.13 (0.8, 1.6) 800 m: 1.04 (0.7, 1.5) | Elemental carbon attributable to traffic, household income, and community deprivation index. |  |
| Hsieh et al. (2019) | Residential Greenness (NDVI was differentiated into the following five exposure levels: 0–20%, 21–40%, 41–60%, 61–80%, and 81–100%). | Asthma incidence | Logistic regression model | OR and 95% CIs for asthma occurrence according to the level of NDVI: 0–20%: Reference 21–40%: 1.17 (1.06–1.30) 41–60%: 1.28 (1.12–1.46) 61–80%: 1.34 (1.15–1.56) 81–100%: 1.10 (0.92–1.32) p for trend: 0.0289 | Urbanization level, frequency of healthcare providers visits, mean township family income, NO_2_, and PM_2.5_. |  |
| Hu et al. (2022) | Residential Greenness (the average annual mean values of NDVI or EVI surrounding the child's home during 2016–2018) | Current asthma was defined according to the ISAAC questionnaire | Logistic regression model | An IQR increase in mean NDVI-250 m (OR):  0.82 (0.72, 0.94)  An IQR increase in mean NDVI-500 m (OR):  0.82 (0.74, 0.93)  An IQR increase in mean NDVI-1000 m (OR):  0.92 (0.84, 1.03) | Child's age, sex, gestational week, delivery mode, duration of exclusive breastfeeding, miscarriage, SES, residing areas, having plants, passive smoking, child's physical activity, sleeping time, screen exposure time, temperature and PM_1_ |  |
| Idani et al. (2020) | Existence of dense green space in a radius of 200 m to the residence | The ECRHS questionnaire was used to examine the presence of asthma. | Logistic regression model | Existence of green space (OR): Yes: Reference No: 1.69 (1.30, 2.20) | Residence in urban areas, having a family history of asthma, having airway hyper responsiveness, the location of the house in the main street with respect to the auxiliary road, the presence of mold in the building, the presence of dense green space in the 200-m radius of the residential home and home gardening. |  |
| Ihlebæk et al. (2017) | Urban green space (VCG and LUG) | The definition of asthma is based on an affirmative answer to the question: “Do you have any of these illnesses, or have you suffered from them in the past?" | Logistic regression model | Highest quintile of NDVI to lowest (OR, men/women): VCG: 0.94 (0.51, 1.74) / 0.81 (0.51, 1.3) LUG: 0.73 (0.4, 1.35) / 0.78 (0.5, 1.23) | Age, ethnicity, education, civil status, use of alcohol, smoking status, physical activity, type of work, number of negative life events, number of good friends and degree of interest from other people, mean income, percentage living in a house that they owned and mean education. |  |
| Kim et al. (2020) | The amount of green areas (m^2^) per capita in 200 administrative regions was obtained from the Korean Statistical Information Service (KOSIS). | AD and AR were respectively assessed by 2 questions on physician diagnosis and current treatment: 1) Have you ever been diagnosed with AD or AR by a physician? 2) Are you currently being treated for AD or AR? Subjects responded as dichotomous variables (“yes” or “no”). | Logistic regression model | For physician-diagnosed atopic dermatitis (OR): Highest quartiles to lowest:  0.82 (0.73, 0.91); For physician-diagnosed allergic rhinitis (OR): 0.94 (0.89, 0.99). | Age, sex, marriage, education, monthly income, and job categories, smoking and alcohol, physical activity and self-reported stress, urbanity and body mass index. |  |
| Kim et al. (2021) | 1) The percent of census tract areas occupied by garden/landscape space of urban land use types including residential, commercial, office, and industrial (%); 2) The percent of census tract areas occupied by greenspace, which is largely accessible by the public, including parks and recreational areas (%); 3) The percent of census tract areas occupied by greenspace, which is not accessible by the public, including golf courses, educational facilities, and cemeteries, as well as agricultural lands (%). | The emergency department visits for asthma per 10,000 people by census tract (patients’ residential location basis, 3-year averages between 2011 and 2013). | The spatial lag model (SLM) and the spatial error model (SEM). | SLM (Coefficient and *p*-value) PrvtGrn: -12.998, *p*>0.10 GrnRec: 3.876, *p*>0.10 SemiGrn: -1.567, *p*>0.10 | Several sociodemographic characteristics at the geographic level were defined as control variables. |  |
| Kuiper et al. (2020) | Greenness (mean NDVI in a circular 100 m, 300 m, 500 m and 1000 m buffer around each participant’s residential address). Mean annual exposures to air pollutants and greenness were averaged across the period 0–18 years of age for parents and 0–10 years of age for offspring. | Offspring early-onset asthma and hay fever were defined as affirmative answers to the questions “For each of your biological children, please tick yes if they have had asthma before 10 years of age”, and “For each of your biological children please tick yes if they have had hay fever/rhinitis”, respectively. | Logistic regression model | NDVI-100m, 300 m, and 500m were not associated with any outcomes, neither in the maternal nor the paternal line. Protective association (OR) of high paternal NDVI exposure (1000 m) on early-onset asthma in offspring: 0.33 (0.14-0.79) | O_3_, NO, grandparental education, grandparental asthma. |  |
| Kuiper et al. (2021) | Residential greenness (mean NDVI in a circular 100 m, 300 m, 500 m and 1000 m buffer around each participant’s residential address). Susceptibility windows (0–10 years, 10–18 years, lifetime, and also separately for one year prior to participation). | Physician diagnosed asthma (ever/allergic/non-allergic), asthma attack last 12 months, current rhinitis |  | Association between lifetime greenness exposures and allergic asthma / rhinitis (OR): NDVI-300 m per 0.1-unit increase: 1.00 (0.98, 1.02) / 1.01 (0.92, 1.11) | O3, NO2, parental asthma and education |  |
| Kwon et al. (2019) | The average NDVI values were calculated for each district. | The number of clinic visits for AR is derived from the National Health Insurance Service’s 2014 database, which includes the number of clinic visits by AR patients over 20 years of age living in Seoul. | The ordinary least squares (OLS) and spatial lag modeling (SLM) models | OLS (Coefficient and *p*-value) 0.537, *p*<0.05 | The distance from Baengnyeong Island, total traffic, manufacturing employee ratio, age, and income level. |  |
| Lee et al. (2018) | The land cover classification maps (1:25,000) of the Korean Ministry of Environment were used to calculate the amount of green space within different buffer sizes (100 m, 200 m, 300 m, and 500 m) around the maternal residential address. | Information on doctor-diagnosed infantile AD at the age of 6 months was obtained from the Korean version of the International Study on Allergies and Asthma in Childhood (ISAAC). | Logistic regression model | Residential surrounding green space (OR): 100-m: 0.997 (0.993, 1.001) 200-m: 0.996 (0.993, 0.999) 300-m: 0.997 (0.995, 0.999) 500-m: 0.999 (0.998, 1.000) | Maternal age, education, income, body mass index, history of allergy, exposure to secondhand smoke, residential mobility, gestational age, the presence of pets, infant sex, birth weight, season of birth, breastfeeding, mode of delivery, temperature, and humidity. |  |
| Lee et al. (2020) | Residential greenness (This study used MODIS NDVI because a single MODIS NDVI image can cover the entire island of Taiwan). | The frequency of clinical visits for AR: longitudinal Health Insurance Database (LHID2000) was used to diagnose children with AR (ICD-9-CM 477, 4770, 4778, 4779). | Generalized additive mixed model | A one unit increase in NDVI (RR): 1.082 (1.040, 1.126) | Air temperature, relative humidity, PM_2.5_ concentrations, socioeconomic status, road network, industrial area, population size, sex ratio, year, season, township urbanization level, and spatial-temporal autocorrelation |  |
| Lee et al. (2022) | The percent of grassland/herbaceous land cover was included by using 2011 National Land Cover Database. | Pediatric asthma emergency department visit/hospitalization rates | Negative binomial regression models | Every 10-percentage point increase in tree canopy cover was expected to bring a 4% decrease in asthma ED visit/hospitalization rates. | The proportion of the Black population residing in census tracts, ozone, toxic facilities rate and road density, population density, vacant housing rates, the rate of housing units built, and open space size rate. |  |
| Li et al. (2019) | Residential greenness (mean NDVI and distance to the nearest park at 100 m, 200 m, 500 m, and 1000 m areas of residential address) | Questions on diagnosis of asthma, pneumonia, rhinitis, and eczema were based on the China, Children, Home and Health study, which used questions adapted from the previously validated International Study of Asthma and Allergies in Childhood (ISAAC) questionnaire. | Logistic regression model | Association between outcomes and NDVI IQR in different buffer areas (OR): Current asthma: 100-m: 1.10 (0.93, 1.29) 200-m: 1.09 (0.86, 1.38) 500-m: 1.01 (0.77, 1.31) 1000-m: 1.02 (0.80, 1.31) Ever asthma: 100-m: 0.97 (0.91, 1.04) 200-m: 0.96 (0.88, 1.06) 500-m: 0.94 (0.84, 1.05) 1000-m: 0.95 (0.86, 1.05) Ever rhinitis: 100-m: 0.96 (0.90, 1.04) 200-m: 0.95 (0.86, 1.06) 500-m: 0.93 (0.82, 1.06) 1000-m: 0.96 (0.86, 1.07) Ever eczema: 100-m: 1.01 (0.94, 1.09) 200-m: 1.01 (0.91, 1.13) 500-m: 1.04 (0.91, 1.19) 1000-m: 1.03 (0.92, 1.16) | Child’s age and sex, ETS at home, parental education, and parental history of asthma |  |
| Lin et al. (2022) | Residential greenness (mean NDVI value within buffer radiuses of 250 m and 500 m around the geocoded residence address). NDVI in pregnancy, postnatal 2 years, and the first 1000 days of life was obtained | Eczema, atopic dermatitis, urticaria, allergic rhinitis (AR), allergic conjunctivitis, food allergy, and asthma from birth to age 2 years | Generalized linear models with a logit link function | Association between per 0.1 units increase in NDVI-500 m and eczema/urticaria/food allergy/AR in the first 1000 days of life (OR): Pregnancy: 1.28 (1.04–1.59) / 1.01 (0.66–1.49) / 0.94 (0.60–1.45) / 1.09 (0.80–1.47) 0–2 years: 1.24 (1.01–1.54) / 0.94 (0.62–1.41) / 0.93 (0.58–1.44) / 1.08 (0.79–1.47) The first 1000 days: 1.26 (1.02–1.56) / 0.96 (0.63–1.43) / 0.93 (0.58–1.45) / 1.09 (0.79–1.48)  Pregnancy NDVI 250-m 3^rd^ versus 1^st^ tertile (eczema/urticaria/food allergy/AR, OR):  1.16 (0.72, 1.88) / 0.70 (0.26, 1.79) / 0.63 (0.24, 1.62) / 0.99 (0.45, 2.16)  Pregnancy NDVI 250-m 2^nd^ versus 1^st^ tertile (OR):  1.00 (0.61, 1.63) / 0.90 (0.37, 2.19) / 0.40 (0.13, 1.12) /1.74 (0.87, 3.58) | Maternal age, maternal employment status, maternal education level, monthly household income, child’s sex, birth season, physical activity in pregnancy, and PM_2.5_ during pregnancy |  |
| Lovasi et al. (2008) | Street tree density (total number of trees on street segments within the UHF divided by land area) | Prevalence of asthma among children aged 4–5 years and hospitalisations for asthma among children less than 15 years old. | Poisson regression models | RR per SD of tree density Prevalence of asthma: 0.71 (0.64, 0.79) Hospitalisations as a result of asthma: 0.89 (0.75, 1.06) | Sociodemographic characteristics, population density and proximity to pollution sources |  |
| Lovasi et al. (2013) | Urban tree canopy coverage (combined LiDAR data and colour infrared aerial imagery) for address at time of birth (250 m). | Current asthma and rhinitis (assessed by BQR questionnaire) at 5 and 7 years old. | Logistic regression model | Relative risk (RR) increase per SD of tree canopy coverage: Asthma at age 5 years: 1.11 (0.85, 1.45) Asthma at age7 years: 1.17 (1.02, 1.33) Rhinitis at age 5 years: 1.60 (0.79, 3.22) Rhinitis at age 7 years: 1.40 (0.63, 3.08) | Sex, age, ethnicity, maternal asthma, previous birth, other previous pregnancy, medicaid, tobacco smoke in home, active maternal smoking, population density, % poverty, % park land, and estimated traffic volume. |  |
| Markevych et al. (2020) | Mean NDVI was calculated for circular buffers of 100, 300, 500 and 1000 m around each participant’s home address at birth. | Data on parent-reported doctor diagnosis of asthma and AR were collected at ages 0.5, 1, 1.5, 2, 4, 6, 10 and 15. Allergic sensitization to food allergens was defined as IgE >0.35 kU/L for allergens in the FX5 screening test (milk, peanut, eggs, soya, cod and wheat flour) at all timepoints. | Generalized estimating equations (GEE) with logit link | Associations between NDVI and asthma / AR up to 15 years (OR):  3^rd^ versus 1^st^ tertile: 100-m: 0.77 (0.50, 1.20) / 0.92 (0.70, 1.21) 300-m: 0.61 (0.39, 0.95) / 0.77 (0.59, 1.01) 500-m: 0.44 (0.27, 0.72) / 0.90 (0.68, 1.19) 1000-m: 0.50 (0.33, 0.75) / 0.84 (0.64, 1.10)  2^nd^ versus 1^st^ tertile:  100-m: 1.10 (0.74, 1.64) / 1.04 (0.79, 1.36)  300-m: 1.20 (0.82, 1.75) / 1.01 (0.78, 1.32)  500-m: 1.46 (1.02, 2.10) / 1.26 (0.96, 1.65)  1000-m: 0.74 (0.50, 1.11) / 1.11 (0.84, 1.46) | Age, sex, family history of allergic diseases, parental education, and season of birth |  |
| Parmes et al. (2020) | Land-cover exposures within a 500 m buffer centred on each child's residential address were computed using data from the Coordination of Information on the Environment (CORINE) program. | Lifetime asthma, current asthma, lifetime allergic rhinitis, and eczema were collected through parental questionnaires. | Logistic regression model | A 10% increase of land covered by green (OR): Lifetime asthma: 1.09 (1.01, 1.18) Current asthma: 1.12 (1.02, 1.23)  AR: 1.08 (1.01, 1.16) Eczema: 1.01 (0.96, 1.06) | Sex, age, BMI, parental history of allergy, maternal education, parental smoking |  |
| Peters et al. (2022) | Environmental greenness (NDVI for five buffer zones around the infant's home address: at the home, 100 m, 500 m, 800 m, and 1600 m radial distances). | IgE-mediated food allergy was defined as a positive OFC in conjunction with a positive test of sensitization. Infants who had a reaction consistent with OFC- stopping criteria in the previous 1 month for egg and 2 months for peanut or sesame were also considered food allergic without completing an OFC. | Mixed effects logistic regression models | Highest tertile of NDVI to lowest (OR): At home: 1.24 (0.96, 1.61) 100-m: 1.40 (1.08, 1.82) 500-m: 1.51 (1.16, 1.96) 800-m: 1.52 (1.17, 1.98) 1600-m: 1.42 (1.09, 1.85) | Parents country of birth, socioeconomic status, dwelling density, pet dog ownership and family history of food allergy. |  |
| Pilat et al. (2012) | Average NDVI per metropolitan statistical area in the months of April - June 2006 | Current asthma rates | - | A Pearson's product–moment correlation for the average NDVI and the residual asthma variable. Reported as not significant. | Relative humidity, temperature, ozone, particulate matter, and ethnicity. |  |
| Putra et al. (2022) | Green space quality was assessed by asking caregivers the extent to which they agreed on the following statements: “There are good parks, playgrounds and play spaces in this neighbourhood.” | Asthma status was determined based on the ISAAC. | Trajectory mixture models | Exposure to quality green space was not associated with the reduced risk of asthma symptoms. | Child’s age, sex, indigenous status, language spoken at home, caregiver education, family income, family structure, number of siblings, area disadvantage, and area accessibility |  |
| Rufo et al. (2021) | Residential greenspace (mean NDVI and SRI at 100, 200, and 300 m radius from the participant's house, measured at birth). | A self-reported questionnaire based on the International Study on Allergy and Asthma Meeting (ISAAC) standardized questionnaire was completed be the participant' legal guardians at the age of 4 and 7 cohort follow-up. Individuals who reported allergy, rhinitis or eczema diagnosed by a physician were regarded as having allergic disease. | Generalized linear mixed models | Highest tercile of NDVI-100 m to lowest (OR): Asthma at age 7: 0.37 (0.12, 1.14) Rhinitis at age 7: 0.37 (0.15, 0.93) | Sex, neighbourhood deprivation, distance to major roads, maternal history of asthma, crowding and maternal education. |  |
| Sbihi et al. (2015) | Surrounding Greenness (NDVI) during the perinatal period. (Measured in 100 m areas around residential postal codes). | Incident asthma during preschool-age (0–5 years); incident asthma during school years (6–10 years). | Nested conditional logistic regression | Per interquartile (0.11) NDVI increase (OR): Pre-school aged: 0.96 (0.93, 0.99)  School aged: 0.99 (0.92, 1.07) | Maternal age at delivery, parity, breastfeeding status at discharge, birth weight, gestational period, household income, and maternal education. |  |
| Sbihi et al. (2016) | Surrounding Greenness (NDVI) during the perinatal period. (measured in 100 m areas around residential postal codes) | Asthma trajectory defined based on group- based trajectory modelling (No asthma, transient, Late-Onset (<3 year) and Early-Onset (<1 year)) | Multinomial logistic regressions | Highest quartile of NDVI to lowest (RR):  Transient: 0.91 (0.80, 1.05)  Late-Onset: 1.05 (0.90, 1.23)  Early-Onset: 1.01 (0.81, 1.25)  Second highest quartile of NDVI to lowest (RR): Transient: 0.98 (0.86, 1.11)  Late-Onset: 1.29 (1.12, 1.49)  Early-Onset: 1.15 (0.94, 1.41) | Gender, parity, breastfeeding initiation, birth weight, delivery mode, maternal smoking, educational attainment, and household income. |  |
| Squillacioti et al. (2019) | NDVI was calculated for all participants within fixed buffers (300 m radius) around their home address. | The prevalence of asthma and asthma-like symptoms was calculated from self-reported data collected by SIDRIA questionnaire. | Logistic regression model | Higher exposure (3^rd^ tertile vs. 1^st^ tertile) to NDVI was associated to significantly lower ORs for asthma: 0.13 (0.02, 0.7). | Age, sex, body mass index (BMI) and urinary cotinine levels. |  |
| Tischer et al. (2017) | Residential surrounding greenness (mean NDVI values in 300-m buffers around the place of residence at the time of birth and at age 4 years were calculated). | Asthma and AR were assessed in a questionnaire at the age of 4 years. | Logistic regression model | NDVI 3^rd^ versus 1^st^ tertile (OR):  Asthma: 1.82 (0.71–4.67) AR: 0.57 (0.22–1.50)  NDVI 2^nd^ versus 1^st^ tertile (OR):  Asthma: 0.82 (0.37–1.81) AR: 0.69 (0.34–1.43) | Sex, cohort, maternal education, maternal smoking during pregnancy, any breastfeeding, season of birth, maternal allergy, pets at home at birth, passive smoking at home at 4 years, and area socioeconomic status |  |
| Winnicki et al. (2022) | Early-life exposure to greenspace (percentage greenspace was assessed in a 2 km buffer around home addresses of individuals). | Individuals with asthma were identified based on hospital contacts for asthma ICD-10 diagnoses or filled prescriptions on asthma medication from 1995 to 2018. A subset of individuals based on hospital contact represents more severe cases. | Cox proportional hazards regression | For medication and hospital diagnosis asthma (HR): High exposure ( >28 % greenspace coverage) versus low exposure (0–5 % greenspace coverage): 1.13 (1.00, 1.28) For hospital diagnosis asthma/more severe cases (HR): High exposure versus low exposure: 0.65 (0.50, 0.84) | Age, sex, parental income and education, and familial disposition to asthma. |  |
| Wu et al. (2021) | Mean NDVI in 500 m buffer of participant’s residence. | Eosinophilic asthma was defined as participant reporting asthma-related medication use and elevated eosinophil count of at least 150 cells per μL | Logistic regression model | Highest quartile of NDVI to lowest (OR): 0.94 (0.88, 0.99) | Age, sex, BMI, highest educational qualification, smoking, alcohol drinking, employment status, ethnicity, social activities, and season. |  |
| Yu et al. (2021) | The participant's school was used as the study site with a circular buffer zone of 800 m and 1000 m. | The Chinese version of the Epidemiologic Standardization Project Questionnaire of the American Thoracic Society (ATS-DLD-78-A) was used to collect data on doctor-diagnosed asthma and current asthma. | Logistic mixed-effects regression models | Per IQR increase in NDVI-1000m (OR): Doctor-diagnosed asthma: 0.77 (0.74-0.80) Current asthma: 0.78 (0.73-0.84) | Age, sex, parental education, family income, obesity, pet kept in home, and exercise time. |  |
| Zeng et al. (2020) | Greenness surrounding schools was defined as the mean of NDVI or SAVI values in circular buffers of 100 m, 300 m, 500 m, and 1000 m around each school. | Information on current asthma was collected using the validated Chinese version of Epidemiologic Standardization Project Questionnaire of the American Thoracic Society. | Logistic mixed-effects regression models | Per 0.1-unit increase of NDVI (OR):  100-m: 0.94 (0.89, 0.98)  300-m: 0.87 (0.82, 0.92)  500-m: 0.82 (0.77, 0.87)  1000-m: 0.81 (0.75, 0.86) | Age, gender, parental education, family income, breastfeeding, low birthweight, preterm, residential area, SHS, mould in home, home coal usage, and family history of asthma. |  |

**Table S4.** Newcastle-Ottawa quality assessment scale for cohort studies

| **Cohort** | **Selection** | | | | **Comparability** | **Outcome** | | | Quality score |
| --- | --- | --- | --- | --- | --- | --- | --- | --- | --- |
|  | Representativeness of the exposed cohort | Selection of the non exposed cohort | Ascertainment of exposure | Demonstration that outcome of interest was not present at start of study | Comparability of cohorts on the basis of the design or analysis | Assessment of outcome | Was follow-up long enough for outcomes to occur | Adequacy of follow up of cohorts |  |
| Lin et al. (2022) | **★** | **★** | **★** | **★** | **★** | **★** | - | **★** | 8 |
| Winnicki et al. (2022) | **★** | **★** | **★** | **★** | **★** | **★** | **★** | **★** | 8 |
| Hartley et al. (2022) | high-risk atopic children | **★** | **★** | **★** | - | **★** | **★** | **★** | 6 |
| Putra et al. (2022) | **★** | **★** | **★** | **★** | - | parental report | **★** | **★** | 6 |
| Cilluffo et al. (2022) | asthmatic children | **★** | **★** | **★** | - | **★** | **★** | **★** | 6 |
| Rufo et al. (2021) | **★** | **★** | **★** | **★** | **★** | parental report | **★** | **★** | 7 |
| Markevych et al. (2020) | **★** | **★** | **★** | **★** | **★★** | parental report | **★** | **★** | 8 |
| Lee et al. (2020) | children diagnosed with AR | **★** | **★** | **★** | - | **★** | **★** | **★** | 6 |
| Kuiper et al. (2020) | **★** | **★** | **★** | **★** | **★** | parental report | **★** | **★** | 7 |
| Gernes et al. (2019) | high-risk atopic children | **★** | **★** | **★** | - | **★** | **★** | **★** | 6 |
| Lee et al. (2018) | **★** | **★** | **★** | **★** | **★★** | parental report | **★** | **★** | 8 |
| Donovan et al. (2018) | **★** | **★** | **★** | **★** | **-** | **★** | **★** | **★** | 7 |
| Tischer et al. (2017) | **★** | **★** | **★** | **★** | **★★** | parental report | **★** | **★** | 8 |
| Sbihi et al. (2016) | **★** | **★** | **★** | **★** | - | **★** | **★** | **★** | 7 |
| Brokamp et al. (2016) | high-risk atopic children | **★** | **★** | **★** | - | **★** | **★** | **★** | 6 |
| Fuertes et al. (2016) | **★** | **★** | **★** | **★** | **★** | parental report | **★** | **★** | 7 |
| Sbihi et al. (2015) | **★** | **★** | **★** | **★** | - | **★** | **★** | - | 6 |
| Fuertes et al. (2014) | **★** | **★** | **★** | **★** | **★** | parental report | **★** | **★** | 7 |
| Lovasi et al. (2013) | **★** | **★** | **★** | **★** | **★** | parental report | **★** | **★** | 7 |

**Table S5.** Quality assessment of cross-sectional studies included in the systematic review

| **Study** | **Questions of the Quality Assessment Tool for Cross-Sectional Studies** | | | | | | | **Overall rating** |  |
| --- | --- | --- | --- | --- | --- | --- | --- | --- | --- |
|  |  |  |  |  |  |  |  |  |  |
|  | **1** | **2** | **3** | **4** | **5** | **6** | **7** |  |  |
| Peters et al. (2022) | √ | √ | √ | √ | √ | √ | √ | Good |  |
| Lee et al. (2022) | **-** | √ | √ | √ | √ | √ | √ | Fair |  |
| Dzhambov et al. (2021) | √ | √ | √ | √ | √ | √ | √ | Good |  |
| Kim et al. (2021) | √ | √ | √ | √ | √ | √ | √ | Good |  |
| Donovan et al. (2021) | √ | √ | √ | √ | √ | √ | √ | Good |  |
| Wu et al. (2021) | - | √ | √ | √ | √ | √ | √ | Fair |  |
| Yu et al. (2021) | √ | √ | √ | √ | √ | √ | √ | Good |  |
| Dong et al. (2021) | - | √ | √ | √ | √ | √ | √ | Fair |  |
| Parmes et al. (2020) | √ | √ | √ | √ | √ | √ | √ | Good |  |
| Kim et al. (2020) | √ | √ | √ | √ | √ | √ | √ | Good |  |
| DePriest et al. (2020) | - | √ | √ | √ | √ | √ | √ | Fair |  |
| Idani et al. (2020) | √ | √ | × | √ | √ | √ | √ | Fair |  |
| Zeng et al. (2020) | √ | √ | √ | √ | √ | √ | √ | Good |  |
| Douglas et al. (2019) | √ | √ | √ | √ | √ | √ | √ | Good |  |
| Li et al. (2019) | √ | √ | √ | √ | √ | √ | √ | Good |  |
| Kwon et al. (2019) | √ | √ | √ | √ | √ | √ | √ | Good |  |
| Squillacioti et al. (2019) | √ | √ | √ | √ | √ | √ | √ | Good |  |
| Eldeirawi et al. (2018) | √ | √ | √ | √ | √ | √ | √ | Good |  |
| Alcock et al. (2017) | √ | √ | √ | √ | √ | √ | √ | Good |  |
| Ihlebæk et al. (2017) | × | √ | √ | √ | √ | √ | √ | Fair |  |
| Dadvand, et al. (2014) | √ | √ | √ | √ | √ | √ | √ | Good |  |
| Fuertes et al. (2014) | √ | √ | √ | √ | √ | √ | √ | Good |  |
| Pilat et al. (2012) | √ | √ | √ | × | √ | √ | × | Poor |  |
| Lovasi et al. (2008) | √ | √ | √ | √ | √ | √ | √ | Good |  |
| Hu et al. (2022) | √ | √ | √ | √ | √ | √ | √ | Good |  |

1. Was the participation rate of eligible persons at least 50%?

2. Were all the subjects selected or recruited from the same or similar populations (including the same time period)? Were inclusion and exclusion criteria for being in the study prespecified and applied uniformly to all participants?

3. For exposures that can vary in amount or level, did the study examine different levels of the exposure as related to the outcome (e.g., categories of exposure, or exposure measured as continuous variable)?

4. Were the exposure measures (independent variables) clearly defined, valid, reliable, and implemented consistently across all study participants?

5. Were the outcome measures (dependent variables) clearly defined, valid, reliable, and implemented consistently across all study participants?

6. Were the outcome assessors blinded to the exposure status of participants?

7. Were key potential confounding variables measured and adjusted statistically for their impact on the relationship between exposure(s) and outcome(s)?

**Table S6.** Newcastle-Ottawa quality assessment scale for case-control studies

| **Case-control** | **Selection** | | | | **Comparability** | **Exposure** | | | Quality score |
| --- | --- | --- | --- | --- | --- | --- | --- | --- | --- |
|  | Is the case definition adequate? | Representativeness of the cases | Selection of Controls | Definition of Controls | Comparability of cases and controls on the basis of the design or analysis | Ascertainment of exposure | Same method of ascertainment for cases and controls | Non-Response rate |  |
| De Roos et al. (2022) | **★** | **★** | - | **★** | - | **★** | **★** | **★** | 6 |
| Kuiper et al. (2021) | self-reported | **★** | **★** | **★** | **★** | **★** | **★** | **-** | 6 |
| Hsieh et al. (2019) | according to the ICD-9-CM | **★** | - | **★** | - | **★** | **★** | **★** | 5 |
| Andrusaityte et al. (2016) | parental report | **★** | **★** | **★** | **★** | **★** | **★** | **★** | 7 |
